# Mutations of 1p genes do not consistently abrogate tumor suppressor functions in 1p-intact neuroblastoma

**DOI:** 10.1186/s12885-022-09800-0

**Published:** 2022-06-30

**Authors:** Chik Hong Kuick, Jia Ying Tan, Deborah Jasmine, Tohari Sumanty, Alvin Y. J. Ng, Byrrappa Venkatesh, Huiyi Chen, Eva Loh, Sudhanshi Jain, Wan Yi Seow, Eileen H. Q. Ng, Derrick W. Q. Lian, Shui Yen Soh, Kenneth T. E. Chang, Zhi Xiong Chen, Amos H. P. Loh

**Affiliations:** 1 Department of Pathology and Laboratory Medicine, KK Women’s and Children’s Hospital, Singapore, 229899 Singapore; 2 Neurodevelopment and Cancer Laboratory, NUS Center for Cancer Research, Yong Loo Lin School of Medicine, National University of Singapore, Singapore, 117599 Singapore; 3 Department of Physiology, Yong Loo Lin School of Medicine, National University of Singapore, Singapore, 117597 Singapore; 4 Comparative and Medical Genomics Laboratory, Institute of Molecular and Cell Biology, A*STAR, Singapore, 138673 Singapore; 5 VIVA-KKH Paediatric Brain and Solid Tumour Programme, Children’s Blood and Cancer Centre, KK Women’s and Children’s Hospital, Singapore, 229899 Singapore; 6 Department of Paediatric Subspecialties Haematology Oncology Service, KK Women’s and Children’s Hospital, Singapore, 229899 Singapore; 7 Duke NUS Medical School, Singapore, 169857 Singapore; 8 National University Cancer Institute, Singapore, 119074 Singapore; 9 Department of Paediatric Surgery, KK Women’s and Children’s Hospital, Singapore, 229899 Singapore

**Keywords:** Neuroblastoma, Chromosome 1, Tumor suppressor, Next-generation sequencing, Synchronous Mutations

## Abstract

**Background:**

Deletion of 1p is associated with poor prognosis in neuroblastoma, however selected 1p-intact patients still experience poor outcomes. Since mutations of 1p genes may mimic the deleterious effects of chromosomal loss, we studied the incidence, spectrum and effects of mutational variants in 1p-intact neuroblastoma.

**Methods:**

We characterized the 1p status of 325 neuroblastoma patients, and correlated the mutational status of 1p tumor suppressors and neuroblastoma candidate genes with survival outcomes among 100 1p-intact cases, then performed functional validation of selected novel variants of 1p36 genes identified from our patient cohort.

**Results:**

Among patients with adverse disease characteristics, those who additionally had 1p deletion had significantly worse overall survival. Among 100 tumor-normal pairs sequenced, somatic mutations of 1p tumor suppressors KIF1Bβ and CHD5 were most frequent (2%) after ALK and ATRX (8%), and BARD1 (3%). Mutations of neuroblastoma candidate genes were associated with other synchronous mutations and concurrent 11q deletion (*P* = 0.045). In total, 24 of 38 variants identified were novel and predicted to be deleterious or pathogenic. Functional validation identified novel KIF1Bβ I1355M variant as a gain-of-function mutation with increased expression and tumor suppressive activity, correlating with indolent clinical behavior; another novel variant CHD5 E43Q was a loss-of-function mutation with decreased expression and increased long-term cell viability, corresponding with aggressive disease characteristics.

**Conclusions:**

Our study showed that chromosome 1 gene mutations occurred frequently in 1p-intact neuroblastoma, but may not consistently abrogate the function of bonafide 1p tumor suppressors. These findings may augment the evolving model of compounding contributions of 1p gene aberrations toward tumor suppressor inactivation in neuroblastoma.

**Supplementary Information:**

The online version contains supplementary material available at 10.1186/s12885-022-09800-0.

## Background

Molecular aberrations of the short arm of chromosome 1 are common and consistent in neuroblastoma, and are well known to correlate with clinical risk, treatment outcomes, as well as other molecular risk markers such as MYCN amplification [1–3]. Inactivation of 1p tumor suppressors through chromosomal deletion is implicated as the key pathogenic mechanism [4, 5]. However, recurrent disease and poor outcome also occur in children with tumors without 1p loss, suggesting that other molecular alterations of 1p candidate genes – particularly tumor suppressors – may contribute to an aggressive disease phenotype in 1p-intact neuroblastoma patients.

Gene mutations play important roles in neuroblastoma disease behavior, particularly involving transcription factors responsible for neurodevelopmental regulation. Germline variants of ALK and PHOX2B are strongly associated with familial predisposition to neuroblastoma [6–8], the latter also associated with congenital central hypoventilation syndrome and Hirschsprung disease. In high-risk neuroblastoma, the commonest somatic variants encountered include ALK, PTPN11, and ATRX [9, 10]. Other mutational variants associated with increased tumor proliferation and poor outcome include LIN28B, BARD1 and LMO1 [10–12]. However, the effect of mutations in other genes like TP73, Let-7 and TIAM1 are less clear with mixed phenotypes observed [13–15]. Additionally, the low mutational burden in neuroblastoma further decreases the opportunities to observe the clinical impact of these variants in patients.

Mutations resulting in functional impairment of key 1p genes may mimic the functional effects of 1p deletion in neuroblastoma. Indeed the down-regulation of genes from this chromosomal region have been observed in familial and high-risk disease, but the mechanism by which this occurs is still not well understood [16, 17]. Most commonly, the expression of genes in 1p are affected by segmental chromosomal aberrations which are common in neuroblastoma, however the incidence and effect of mutational variants affecting these genes is less well studied. Among 1p candidate genes, we previously described the mechanistic role of tumor suppressor KIF1Bβ in the tumorigenesis of neuroblastoma, and its interaction with downstream partners XAF1 and RNA helicase A [18–20], as well as the effects of loss-of-function mutations on expression-mediated apoptosis [21]. Correspondingly, germline loss-of-function mutations of KIF1Bβ have also been implicated in familial predisposition to neuroblastoma [22]. Thus, to understand the incidence, spectrum and effects of mutational variants in 1p-intact neuroblastoma, we developed a targeted sequencing panel of 21 candidate genes that were commonly mutated and prognostically significant in neuroblastoma, and used it to profile the mutational variants in a retrospective cohort of 1p-intact neuroblastoma tumors. We then correlated the identified variants with clinical, cytogenetic and pathological characteristics, and performed functional validation of selected novel variants of 1p36 genes identified from our patient cohort.

## Methods

### Patients and specimens

Neuroblastoma tumor specimens managed at the Department of Pathology and Laboratory Medicine, KK Women’s and Children’s Hospital were accrued. Data on patient demographics, clinical stage and tumor histology were obtained from available clinical metadata. All specimens were evaluated by a consultant pathologist to be representative lesional or normal tissue. Clinical follow-up data was obtained from the Singapore Childhood Cancer Registry, clinical charts and hospital visit records. This study was granted waiver of informed consent by SingHealth Centralized Institutional Review Board (protocols 2012/450, 2014/2079).

### FISH for evaluation of MYCN, 1p and 11q copy number

FISH assays were carried out on formalin-fixed, paraffin-embedded tissue sections (FFPE) slides with MYCN SpectrumGreen and CEP2 SpectrumOrange DNA probes (Abbott Molecular), CHD5/CCP1 FISH probe (CytoTest), KMT2A/CCP11 (CytoTest) and MPO/CEP17 (Kreatech). FFPE slides were baked at 56 °C overnight, and deparaffinized in xylene and 100% ethanol. Enzymatic digestion was carried out with Protease I Solution (Abbott Molecular), DNA probe mixture was applied to the target area and co-denatured, and hybridization carried out overnight at 37 °C. The slides were analyzed under an epifluorescence microscope and captured and processed using Isis software (Metasystems GmbH).

### Targeted next-generation sequencing

Based on literature search and previous experience, 21 significant genes involved in neuroblastoma were identified that were regarded as leading candidate genes associated with worse clinical prognosis, that had been identified through prior clinical sequencing and expression profiling studies (see Additional File 1 (Supplementary Table 1)). Amplified exonic regions were sequenced on an Ion Proton (RRID:SCR_017982) platform to a depth of 500 × coverage. Sequences were aligned to a reference genome and variants called. First, common SNPs with an allele frequency of > 1% according to the 1000 genomes project (1000G) found in local and international populations were filtered out. The remaining variants of the tumor sample were compared with those from normal sample and common variants between the two were regarded as germline. Variants were processed to retain only those that cause a missense codon, stop codon or a frameshift, or an indel. Reported variants were compared against those previously reported in neuroblastoma samples from other populations, and selectively validated with Sanger sequencing.

### Statistical analysis

Patient characteristics and sequencing results were tabulated with means and proportions. Categorical variables were analyzed using chi-square test (asymptotic 2-sided), and survival analysis performed using Kaplan–meier method with log-rank test. *P* < 0.05 was considered significant. Statistical analysis was performed using SPSS v.13.0 (Armonk, NY).

### Cell lines

Neuroblastoma cell lines SK-N-AS (RRID:CVCL_1700) and NLF (RRID: RRID:CVCL_E217) were obtained from American Type Culture Collection (ATCC), and were cultured in RPMI-1640 containing 10% FBS and 1% 2.05 mM L-Glutamine (Hyclone). Both cell lines were maintained at 37 °C in a 5% CO2 humidified incubator. Cell line transfection, transduction and preparation of lentiviral-delivered shRNA are outlined in Additional File 4 (Supplementary methods).

### Immunoblotting

Cells were harvested and lysed with protease inhibitor-containing EBC buffer (50 mM Tris pH 8.0, 120 mM NaCl, 0.5% NP-40). After lysing, cells were centrifuged at 4 °C. The resulting supernatant was removed prior to quantification of protein concentration using Bradford Assay. Laemmli buffer and β-Mercaptoethanol was added to equal amounts of protein in cell lysates. Protein samples of 100 – 200 μg were subsequently separated with 12% and 8% Sodium Dodecyl Sulfate polyacrylamide gel electrophoresis before transfer to PVDF membrane (Bio-Rad). Primary antibodies were added overnight following blocking in 5% milk in PBS-T, and were as follows: rabbit His-tag antibody (Cell Signalling Technology, RRID:AB_2115720), mouse CHD antibody (Santa Cruz Biotechnology, RRID:AB_10610044), mouse monoclonal p16INK4a antibody (BD Pharmingen, RRID:AB_394077), mouse monoclonal β-actin (Santa Cruz Biotechnology, RRID:AB_2833259), mouse FLAG-tag antibody (Sigma-Aldrich) and rabbit cleaved caspase-3 (CC3) antibody (Cell Signalling Technology). Visualisation was conducted using iBright1500 (Thermo Fisher). Bands were analysed using ImageJ software and normalized to β-actin.

### Crystal violet colony formation assay

After 24 h transduction, cells were plated in 6-well plates and cultured in medium using puromycin (Gibco) concentrations as previously mentioned. Fresh medium was replenished every 3–4 days. After 1.5–2 weeks, wells were washed with phosphate-buffered saline (PBS) once before crystal violet staining for 30 min. Excess stain was removed via washing repeatedly using Milli-Q water. From scanned images of the wells, the percentage surface area covered by crystal violet stained cell colonies, and their respective staining intensities (representing cell density) were quantified using the ColonyArea plugin on ImageJ, as described [23].

## Results

### Patient and disease characteristics

**Fig. 1:**
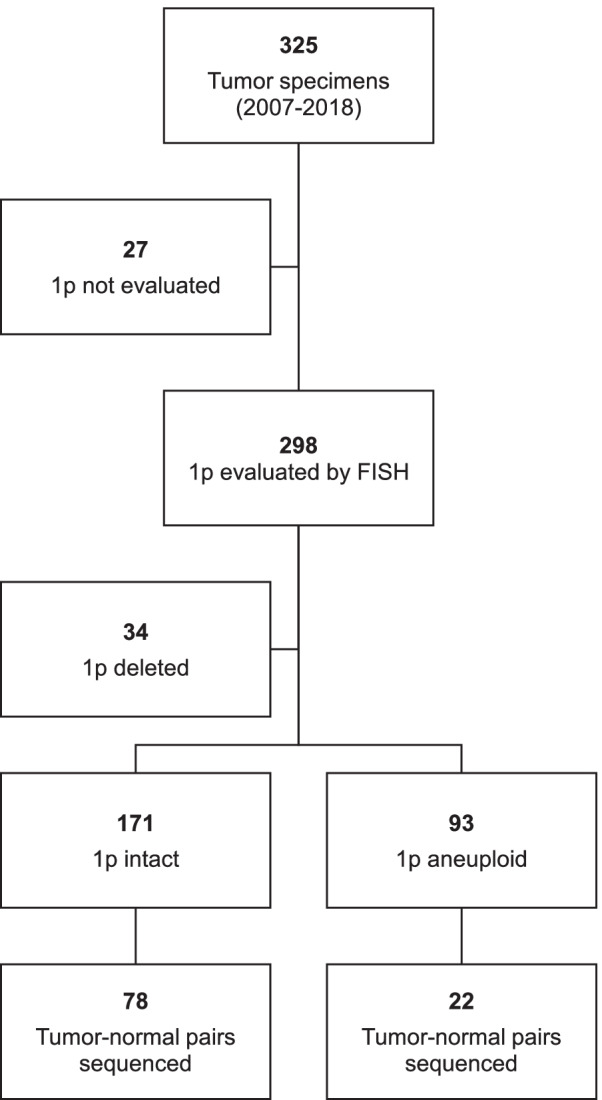
CONSORT diagram of 325 tumor specimens and subgroups evaluated by FISH for 1p status and evaluated using targeted sequencing panel for mutational profile

From 2007–2018, 325 neuroblastoma tumor specimens were managed at the Department of Pathology and Laboratory Medicine, KK Women’s and Children’s Hospital, representing over 90% of all neuroblastoma tumor specimens in Singapore (Fig. 1). Median age of patients was 3.4 years (range: 0.0–13.58), 183 (56.3%) were male, 142 (43.7%) were female. Disease was staged as INSS 1, 2, 3, 4 and 4S in 17 (5.2%), 18 (5.5%), 59 (18.2%), 209 (64.3%) and 2 (0.6%) cases, respectively. Correspondingly, Children’s Oncology Group (COG) risk was assigned as low in 27 (8.3%), intermediate in 38 (11.7%) and high in 241 (74.2%) cases. MYCN was amplified in 75 (23.1%) and not amplified in 214 (65.9%) cases; 28 (8.6%) cases were indeterminate.

### Association of 1p copy number status with known prognostic variables indicates additional factors may be responsible for poor outcomes among 1p-intact patients

**Fig. 2:**
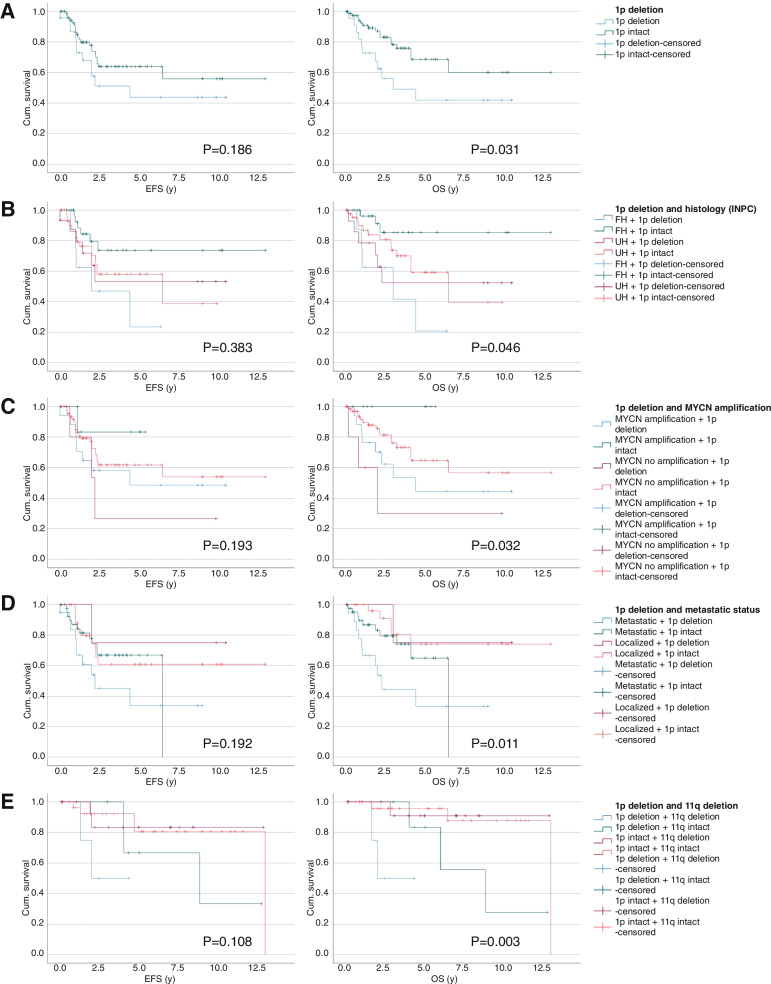
Survival analysis of 1p deletion. Kaplan–meier curves indicating event-free and overall survival according to (**A**) 1p deletion status, (**B**) 1p deletion and MYCN amplification status, (**C**) 1p deletion and INPC histology status, (**D**) 1p deletion and metastatic disease status, (**E**) 1p deletion and 11q deletion status

Copy number status of 1p could be evaluated in 298 of 325 patients: 1p was deleted in 34 (11.4%), intact in 171 (57.4%) and indeterminate or aneuploid in 93 (31.2%) (Fig. 1). Comparing the 205 patients with evaluable 1p status, cases with 1p deletion were associated with established prognostic factors MYCN amplification, metastatic disease and COG high risk status (*P* < 0.001, *P* = 0.065, and *P* = 0.012, respectively, Table 1). Deletion of 1p was significantly associated with lower overall survival (OS) (mean survival 5.4 ± 1.0 y, *P* = 0.031), but not event-free survival (EFS) (mean survival 5.5 ± 1.0 y) (Fig. 2A). Among patients with MYCN amplification, 11q deletion, unfavorable histology and metastatic disease, those who additionally had 1p deletion were associated with significantly worse OS (*P* = 0.046, 0.032, and 0.011, respectively, Fig. 2B-D). However, 1p-intact patients still only had 56% EFS (mean survival 8.3 ± 0.8 y) and 60% OS (mean survival 9.0 ± 0.9 y) (Fig. 2A). While this verified the known phenomenon of poor survival in our patients with 1p deletion, yet the poor survival in those without this risk factor suggested a role of other variables contributing to disease outcomes in 1p-intact patients.

**Table 1:** Distribution of clinical and pathological variables among 205 neuroblastoma cases with and without 1p deletion

Variable		1p deletion (n(%))	1p intact (n(%))	Chi-square	*P*-value
Gender (*n* = 205)	Male	17 (50.0)	103 (60.2)	1.224	0.269
	Female	17 (50.0)	68 (39.8)		
INPC histology (*n* = 204)	FH	11 (32.4)	82 (48.2)	2.881	0.090
	UH	23 (67.6)	88 (51.8)		
Treatment status (*n* = 204)	Pre-chemotherapy	7 (20.6)	33 (19.4)	0.025	0.875
	Post-chemotherapy / relapse	27 (79.4)	137 (80.6)		
Specimen site (*n* = 205)	Primary tumor	30 (88.2)	158 (92.4)	2.104	0.349
	Metastatic tumor	4 (11.8)	10 (5.8)		
	Both	-	3 (1.8)		
MYCN (*n* = 205)	Amplified	25 (73.5)	26 (15.2)	51.623	< 0.001
	Non-amplified	9 (26.5)	145 (84.8)		
11p (*n* = 160)	Deleted	8 (36.4)	34 (24.6)	1.348	0.246
	Not deleted	14 (63.6)	104 (75.4)		
17q (*n* = 45)	Gain	11 (91.7)	21 (63.6)	3.366	0.067
	No gain	1 (8.3)	12 (36.4)		
Metastatic status (*n* = 193)	Metastatic	27 (79.4)	100 (62.9)	3.397	0.065
	Localized	7 (20.6)	59 (82.4)		
COG risk (*n* = 198)	High	32 (94.1)	122 (74.4)	6.341	0.012
	Low/intermediate	2 (5.9)	42 (25.6)		

*INPC* International Neuroblastoma Pathology Classification, *FH* Favorable histology, *UH* Unfavorable histology, *COG* Children’s Oncology Group

### Profiling of mutations in neuroblastoma candidate genes demonstrates high frequency of variants among chromosome 1p genes

**Fig. 3:**
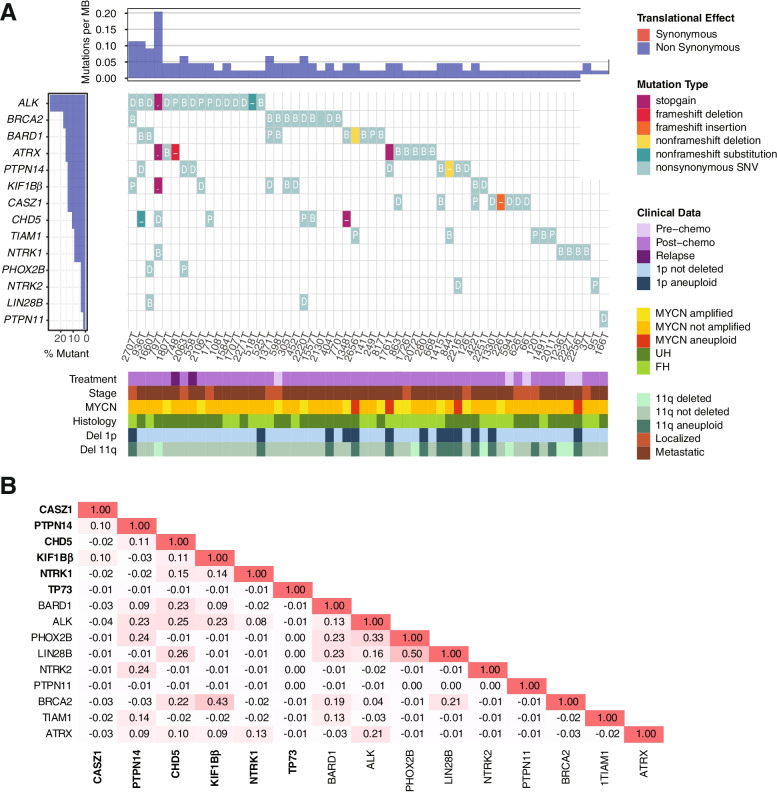
Mutational profile of gene variants in 1p-intact neuroblastoma. **A** Waterfall plot of the distribution of mutations found in 56 neuroblastoma tumors, reflecting mutation type predicted according to Sift in each identified variant (main plot), frequency of mutations per sample (upper panel), frequency of samples mutated (left panel) and clinical-pathological variables (lower panel heatmap). UH: unfavorable histology, FH: favorable histology. **B** Correlation matrix of 15 genes with identified variants in 100 sequenced tumor-normal pairs; names of chromosome 1p genes in bold

To first profile the mutational variants in our neuroblastoma patients, we evaluated pairs of tumor specimens and adjacent normal tissue with a custom targeted sequencing panel covering neuroblastoma candidate genes located at 1p and at other chromosomal loci (see Supplementary Dataset File). In total, 100 tumor-normal pairs were suitable for sequencing analysis (Fig. 1). Among these, 104 non-synonymous variants were identified, the most frequent being ALK (*n* = 14), ATRX (*n* = 10), BARD1 and BRCA2 (*n* = 9 each) (Fig. 3A, Additional File 1 (Supplementary Table 1)). Variants were not detected in MIR34A, SDHB, LMO1, and Let7a-1 / 2 / 3. Highest somatic mutation frequencies were observed in ALK (8%); ATRX and BARD1 (3%), and CHD5 and KIF1Bβ (2%) – both on chromosome 1. Despite filtering for benign population SNPs in the variant calling pipeline, repetitive germline variants were identified: CASZ1 T157M (*n* = 2), CHD5 T157M (*n* = 2), NTRK1 P407L (*n* = 2), TIAM1 S965C (*n* = 2), ATRX F847S (*n* = 4), however their pathogenicity had not been previously determined.

Among 22 patients with multiple synchronous mutations, the most common involved ALK (*n* = 10), BRCA2 (*n* = 8), and chromosome 1 genes PTPN14 (*n* = 7), KIF1Bβ (*n* = 7) and CHD5 (*n* = 6) (Fig. 3B). Together, the high somatic mutation frequencies and associations with other variants suggested a prominent role for mutations of chromosome 1 genes in the clinical spectrum of neuroblastoma, in addition to those of more established gene candidates like ALK and ATRX.

### Cases of 1p-intact neuroblastomas with candidate gene variants are associated with 11q deletion and synchronous mutations in other genes

We next focused on 1p-intact neuroblastomas and studied the mutational spectrum of neuroblastoma candidate genes (particularly chromosome 1 genes CASZ1, CHD5, PTPN14, KIF1Bβ, NTRK1, and TP73), and their association with clinical prognostic variables. Among 78 1p-intact cases sequenced, neuroblastoma candidate gene variants were identified in 59 cases. The presence of neuroblastoma candidate gene variants was significantly associated with 11q deletion (*P* = 0.045, Table 2), but was not significantly associated with histology, treatment status, metastatic disease status, and COG-risk status.

**Table 2:** Distribution of clinical pathological variables among 78 1p-intact neuroblastoma cases with and without mutational variants

Variable		Neuroblastoma candidate gene variant present (n (%))	Neuroblastoma candidate genes variant absent (n (%))	Chi-square	*P*-value
Gender (*n* = 78)	Male	26 (57.8)	18 (54.5)	0.081	0.776
	Female	19 (42.2)	15 (45.5)		
INPC histology (*n* = 78)	FH	18 (40.0)	19 (57.6)	2.359	0.125
	UH	27 (60.0)	14 (42.4)		
Treatment status (*n* = 78)	Pre-chemotherapy	4 (8.9)	5 (15.2)	0.732	0.392
	Post-chemotherapy/ relapse	41 (91.1)	28 (84.8)		
Specimen site (*n* = 78)	Primary tumor	40 (88.9)	32 (97.0)	5.165	0.076
	Metastatic tumor	5 (11.1)	-		
	Both	-	1 (3.0)		
MYCN status (*n* = 78)	Amplified	9 (20.0)	6 (18.2)	0.041	0.840
	Non-amplified	36 (80.0)	27 (81.8)		
11q status (*n* = 76)	Deleted	7 (36.8)	12 (21.1)	4.017	0.045
	Not deleted	12 (63.2)	45 (78.9)		
Metastatic status (*n* = 76)	Metastatic	34 (75.6)	19 (61.3)	1.770	0.183
	Localized	11 (24.4)	12 (38.7)		
COG risk (*n* = 75)	High	40 (88.9)	22 (73.3)	3.040	0.081
	Low/intermediate	5 (11.1)	8 (26.7)		

*INPC* International Neuroblastoma Pathology Classification, *FH* Favorable histology, *UH* Unfavorable histology, *COG* Children’s Oncology Group

**Fig. 4:**
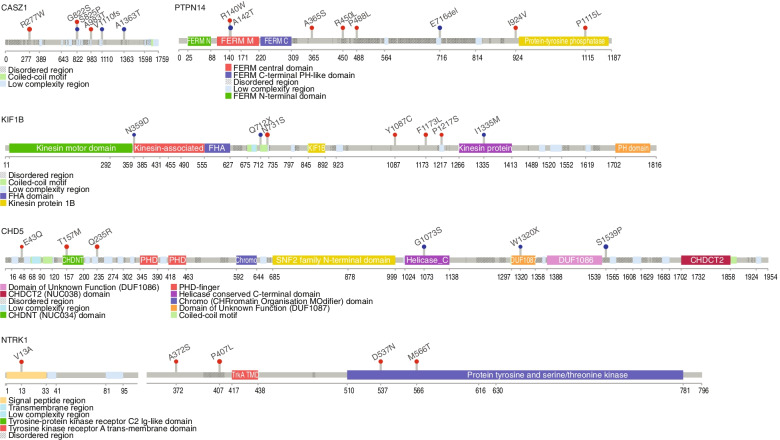
Mutational profile of chromosome 1 genes in 1p-intact neuroblastoma. Lollipop diagrams visually depicting occurrence of identified mutation variants on proteins encoded by the affected 1p genes; red: known variants, blue: novel variants

Among the chromosome 1 candidate genes studied, 38 variants were identified in 29 cases (Fig. 4), with a higher frequency of germline variants compared to other candidate genes. In general, patients with CHD5 (*n* = 6) and PTPN14 (*n* = 8) mutations were older, aged median 4.7 years (range: 2.8–12.3), and 5.6 years (range: 2.7–10.4), respectively, with all patients having metastatic high-risk disease. In contrast, patients with other chromosome 1 gene variants were younger – CASZ1: 3.7 years (range: 1.1–9.4), KIF1B: 3.4 years (range: 1.3–9.4), NTRK1 3.9 years (range: 0.9–5.08), TP73 3.43 years. Synchronous mutations were identified in 6 of 7 patients with CHD5 variants (cases 797 T, 171 T, 2220 T, 1557 T, 936 T, 1348 T), and 7 of 8 patients with PTPN14 variants (844 T, 558 T, 1415 T, 2063 T, 936 T, 2216 T, 1761 T), 7 of 8 patients with KIF1Bβ mutations (797 T, 1371 T, 305 T, 2707 T, 2106 T, 452 T, 422 T), 3 of 8 patients with CASZ1 mutations (1415 T, 863 T, 422 T), and 2 of 5 patients with NTRK1 mutations (797 T, 37 T) Together, this pointed towards an association of adverse clinical characteristics in patients with chromosome 1 gene mutations, most of which were germline variants and tended to occur in association with other concurrent molecular aberrations.

### Novel variants identified among 1p genes are predicted to be pathogenic, while functional validation suggests mixed responses

To understand the potential pathogenic roles of the novel variants we identified, we profiled their predicted variant effects in silico and performed selective functional validation of germline single nucleotide polymorphisms (SNPs) in patients with unique clinical courses. In all, 24 novel variants were found that were not previously described in dbSNP, Clinvar, 1000G and COSMIC databases (Additional File 2 (Supplementary Table 2)). Novel variants predicted by SIFT to be deleterious or pathogenic were identified in PTPTN14 (single nucleotide variant (SNV) A142T in the FERM central domain); CHD5 (SNV G1073S, helicase c-terminal, and SNV E43Q, 5’ UTR); KIF1Bβ (SNV I1335M, kinesin domain); and TIAM1 (SNV D1079N, DH domain). Novel ATRX, BRCA2, NTRK2, BARD1, CASZ1, PHOX2B variants all occurred in coding regions.

**Fig. 5:**
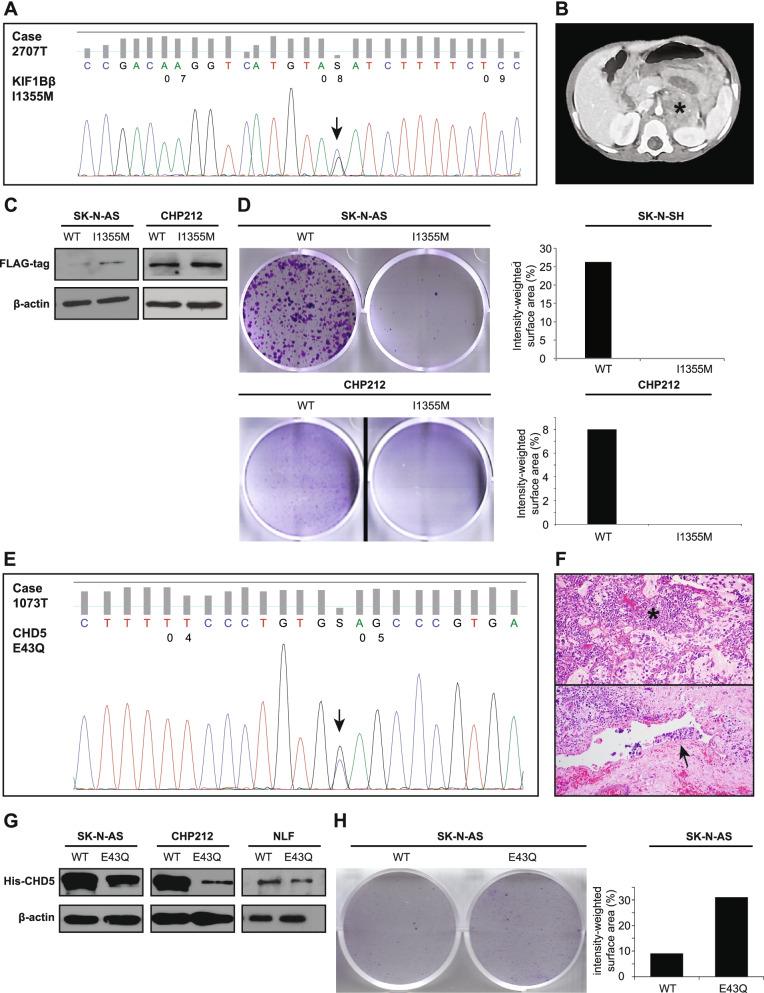
Clinical phenotype and functional validation of novel mutations of KIF1Bβ and CHD5. Characterization of KIF1Bβ I1355M variant in case 2707 T:** A** Sanger sequencing electropherogram, **B** contrast-enhanced CT scan demonstrating persistent but localized retroperitoneal tumor (asterisk), **C** immunoblot analysis and (**D**) crystal violet colony formation assay of wild type (WT) and I1335M mutant SK-N-AS and CHP212 neuroblastoma cells. Characterization of CHD5 E43Q variant in case 1073 T: **E** Sanger sequencing electropherogram, (**F**) photomicrographs of patient tumor demonstrating poorly differentiated neuroblastoma (asterisk) and extensive lymphovascular invasion (arrow) (H&E, 200x), **G** immunoblot of WT and E43Q-transfected SK-N-AS, CHP212 and NLF cells, and (**H**) crystal violet colony formation assay of the former. Western blots cropped and adjusted equally for brightness; full length unadjusted blots are presented in Additional File 3

KIF1Bβ I1335M was identified in a stage 3 intermediate-risk patient with a surprisingly indolent disease course despite multiple synchronous pathogenic mutations including the ALK F1174 hotspot and in BRCA2 (Case 2707 T) (Fig. 5A, B). Overexpression of KIF1Bβ I1335M in SK-N-AS and CHP212 cells increased KIF1Bβ short-term expression, resulting in long-term tumor suppressive activity as indicated by decreased colony formation, compared to KIF1Bβ WT (Fig. 5C, D). A germline missense variant of CHD5 E43Q was identified in a patient with deletion of the WT allele demonstrated on FISH. The patient had multiple poor prognostic features including stage 4 high risk disease, unfavorable histology, extensive lymphovascular invasion and significant residual regional disease after neo-adjuvant chemotherapy with 32 of 33 resected lymph nodes positive for metastatic neuroblastoma (Case 1073 T) (Fig. 5E, F). Overexpression of CHD5 E43Q in SK-N-AS, CHP212 and NLF cells decreased CHD5 short-term expression, compared to CHD5 WT (Fig. 5G). Decreased CHD5 expression due to E43Q corresponded to increased colony formation in SK-N-AS cells, suggesting loss of long-term tumor suppressive activity (Fig. 5H). The functional changes reflected the observed clinical phenotypes in both cases, and suggested that these novel 1p36 variants, while deleterious to tumor suppressors such as CHD5, may contribute to gain-of-function in others such as KIF1Bβ.

## Discussion

Segmental loss of the short arm of chromosome 1p is associated with aggressive disease phenotypes in neuroblastoma. However, it is not known if mutational variants can also inactivate 1p tumor suppressor genes to cause equivalent effects or otherwise. By studying the association of mutational variants of candidate neuroblastoma genes with clinical, cytogenetic and pathological characteristics, and survival outcomes, we found 1p candidate genes CHD5 and KIF1Bβ to be most frequently mutated after ALK, ATRX, and BARD1. Among 78 1p-intact neuroblastoma patients, mutations in 1p genes were often associated with other synchronous mutations and other chromosomal aberrations like 11q deletion. While the novel 1p variants were predicted to be deleterious in silico, functional validation indicated that they did not consistently impair the effect of tumor suppressors KIF1Bβ and CHD5. Taken together, our data disfavors the hypothesis that isolated mutations of 1p candidate genes consistently contribute to loss of tumor suppressor function in 1p-intact patients, but rather demonstrate their varied functional sequelae and propensity to occur in tandem with other molecular aberrations. Our findings lend weight to the evolving idea that 1p tumor suppressor genes may not be primarily inactivated by classic biallelic “two hit” mechanisms, but rather contribute to tumorigenesis via additive dosage-sensitive contributions which may affect multiple components of an antioncogenic interaction cascade or pathway [24, 25]. However, further profiling of other genetic aberrations such as sub-genic deletions and copy number variations in a larger cohort will be required to verify this.

The 1p36 region is thought to harbor potent master tumor suppressor genes, that when inactivated by structural or single nucleotide variations, or by post-transcriptional silencing, would lead to loss of replicative control. Chief candidates have centered on CHD5 and KIF1Bβ [16, 26–28], which we found in our series to be the most frequently mutated genes after ALK and ATRX. However, conflicting evidence argues against the singular role of either of these tumor suppressors. A consistent single region of overlapping heterozygous deletions has not been identified in human cancers and the deleted intervals are often large, especially in advanced cancers [29]. In neuroblastoma, the smallest identifiable region of overlap still amounts to 2 Mb [30]. Thus, it has been proposed instead that combined loss of several 1p36 tumor suppressors may be necessary for tumorigenesis [21], or that this region may be inherently unstable, predisposing it to recurrent chromosomal rearrangements. We observed frequent occurrence of synchronous mutations in patients with 1p gene variants, a phenomenon that has been observed in other cancers associated with mutations of CHD5 [31]. Synchronous mutations were identified in all patients with CASZ1 variants. Although overall mutational frequencies in neuroblastoma are low [32, 33], CASZ1 has not been previously noted to be associated with other gene mutations in neuroblastoma.

CHD5 is preferentially expressed in the testis and nervous system, particularly the nucleus of mature neurons [34], and functions as a transcriptional regulator via chromatin remodeling, binding the N terminus of H3 through its tandem plant homeodomains (PHDs) with dose-dependent effects [26]. CHD5 expression is significantly lower in high-risk neuroblastoma tumors [35], but the role of mutations in modulating its function is poorly understood. Mutations of the H3-binding domains impair the tumor suppressive activity of CHD5 [36]. However, in patient tumors (of prostate and ovarian cancer, and melanoma), prevalence of somatic CHD5 mutations is low [4, 31, 37–39]. Likewise, few somatic CHD5 mutations have been reported in neuroblastoma patients, and only in the context of relapse [40]. Among neuroblastoma cell lines, in only 1 of 30 cell lines, was a V680L heterozygous missense mutation detected – a variant not affecting the H3-binding function of CHD5 [30]. We too did not identify any mutations involving the CHD5 PHD domains in our cases. Instead, pathogenic effects are thought to arise from heterozygous deletion of one gene copy and silencing of the remaining allele or biallelic inactivation via promoter hypermethylation [30, 41]. We illustrate such a patient with a loss-of-function mutation at the 5’ UTR region of the remaining allele and a corresponding aggressive disease course. This clinical case supports the role of CHD5 as a haploinsufficient tumor suppressor in neuroblastoma.

KIF1Bβ is an intracellular motor protein that regulates neuronal differentiation and survival that was also identified as a key tumor suppressor in the 1p36 region [28]. In neuroblastoma, frequency of inactivation is correlated with aggressive disease behavior: hemizygous deletion has been detected in 18% of early-stage tumors, in 55% of patients with stage 3 and 4 disease, and in 84% of MYCN-amplified tumors [42]. Chromosomal deletion appears to be the main mechanism for its inactivation as promoter hypermethylation and somatic mutations of the coding region are infrequently encountered in patient samples and cell lines [5, 16, 42], as we have similarly observed. While loss of KIF1Bβ has been implicated in other neurogenic tumors like paragangliomas and phaeochromocytomas, mutations have also been infrequently encountered in most clinical series of these cancers [22, 43–45], although higher mutational frequencies have been observed in selected populations [46, 47].

In this first focused description of the mutational landscape of neuroblastoma in an Asian population, prevalence of mutations resembled Western series, most commonly involving genes such as ALK and ATRX. The respective mutation frequencies were similar to that of North American neuroblastoma patient populations that were interrogated with whole exome sequencing (WES) (ALK, 8% versus 9.2%; ATRX, 3% vs 2.5%). However, few were recurrent, with only ALK variants R127Q, R1275Q, F1174C and F1174L previously reported in neuroblastoma [9]. Notably, recognized coding variants rs2070094, rs2229571, rs17489363 and rs1048108 were not identified in our cohort [48, 49], the latter specifically recognized as a neuroblastoma susceptibility polymorphism in Han Chinese, which constitute the ethnic majority in Singapore [50]. The effect of such polymorphisms on modulation of disease phenotypes in different ethnic cohorts remains limited due to the scarcity of data from non-Western populations, and warrants further study. In our local population, we previously reported a particularly high incidence of germline variants of uncertain significance among neuroblastomas interrogated with WES. These involved DNA repair genes such as BRCA1, BRCA2, MLH1 and ATRX, but none involving chromosome 1p candidate genes in that smaller cohort [51]. Future studies could explore this group of DNA repair genes, as well as intronic and non-coding SNPs associated with neuroblastoma, which were not tested in this study [48, 50, 52, 53].

## Conclusion

In this large cohort of neuroblastoma patients, CHD5 and KIF1Bβ were the most frequently mutated 1p genes, and were associated with multiple other synchronous mutations. However, the functional effects of these mutations were varied, and may not consistently lead to abrogation of tumor suppressor function akin to 1p deletion. Indeed, the overall tumor phenotype and outcome may be dependent on the balance of effects exerted by variants of respective tumor suppressor genes in 1p-intact neuroblastomas.

## Supplementary Information

**Additional file 1: Supplementary Table 1.** Overview of all mutational variants identified in this study.

**Additional file 2: Supplementary Table 2.** Novel variants not previously described in dbSNP, Clinvar, 1000G, COSMIC databases.

**Additional file 3.** Full length western blots.

**Additional file 4.** Supplementary methods.

## Data Availability

The datasets supporting the conclusions of this article are available in the European Nucleotide Archive, https://www.ebi.ac.uk/ena/browser/view/PRJEB47686.

## Abbreviations

ATCC: American Type Culture Collection
COG: Children’s Oncology Group
EFS: Event free survival
FISH: Fluorescence in-situ hybridization
FFPE: Formalin-fixed, paraffin-embedded
OS: Overall survival
SNP: Single nucleotide polymorphism
SNV: Single nucleotide variant
WES: Whole exome sequencing

## References

[1] Maris JM, Guo C, Blake D, White PS, Hogarty MD, Thompson PM (2001). Comprehensive analysis of chromosome 1p deletions in neuroblastoma. Med Pediatr Oncol.

[2] Spitz R, Hero B, Ernestus K, Berthold F (2003). FISH analyses for alterations in chromosomes 1, 2, 3, and 11 define high-risk groups in neuroblastoma. Med Pediatr Oncol.

[3] Defferrari R, Mazzocco K, Ambros IM, Ambros PF, Bedwell C, Beiske K (2015). Influence of segmental chromosome abnormalities on survival in children over the age of 12 months with unresectable localised peripheral neuroblastic tumours without MYCN amplification. Br J Cancer.

[4] Koyama H, Zhuang T, Light JE, Kolla V, Higashi M, McGrady PW (2012). Mechanisms of CHD5 Inactivation in neuroblastomas. Clin Cancer Res.

[5] Yang HW, Chen YZ, Takita J, Soeda E, Piao HY, Hayashi Y (2001). Genomic structure and mutational analysis of the human KIF1B gene which is homozygously deleted in neuroblastoma at chromosome 1p36.2. Oncogene..

[6] Wang W, Zhong Q, Teng L, Bhatnagar N, Sharma B, Zhang X (2014). Mutations that disrupt PHOXB interaction with the neuronal calcium sensor HPCAL1 impede cellular differentiation in neuroblastoma. Oncogene.

[7] Mossé YP, Laudenslager M, Longo L, Cole KA, Wood A, Attiyeh EF (2008). Identification of ALK as a major familial neuroblastoma predisposition gene. Nature.

[8] Mosse YP, Laudenslager M, Khazi D, Carlisle AJ, Winter CL, Rappaport E (2004). Germline PHOX2B mutation in hereditary neuroblastoma. Am J Hum Genet.

[9] Pugh TJ, Morozova O, Attiyeh EF, Asgharzadeh S, Wei JS, Auclair D (2013). The genetic landscape of high-risk neuroblastoma. Nat Genet.

[10] Lasorsa VA, Formicola D, Pignataro P, Cimmino F, Calabrese FM, Mora J (2016). Exome and deep sequencing of clinically aggressive neuroblastoma reveal somatic mutations that affect key pathways involved in cancer progression. Oncotarget.

[11] Oldridge DA, Wood AC, Weichert-Leahey N, Crimmins I, Sussman R, Winter C (2015). Genetic predisposition to neuroblastoma mediated by a LMO1 super-enhancer polymorphism. Nature.

[12] De Wilde B, Beckers A, Lindner S, Kristina A, De Preter K, Depuydt P (2018). The mutational landscape of. Oncotarget.

[13] Sanmartín E, Yáñez Y, Fornés-Ferrer V, Zugaza JL, Cañete A, Castel V (2017). TIAM1 variants improve clinical outcome in neuroblastoma. Oncotarget.

[14] Powers JT, Tsanov KM, Pearson DS, Roels F, Spina CS, Ebright R (2016). Multiple mechanisms disrupt the let-7 microRNA family in neuroblastoma. Nature.

[15] Romani M, Scaruffi P, Casciano I, Mazzocco K, Lo Cunsolo C, Cavazzana A (1999). Stage-independent expression and genetic analysis of tp73 in neuroblastoma. Int J Cancer.

[16] Carén H, Ejeskär K, Fransson S, Hesson L, Latif F, Sjöberg RM (2005). A cluster of genes located in 1p36 are down-regulated in neuroblastomas with poor prognosis, but not due to CpG island methylation. Mol Cancer.

[17] Maris JM, Kyemba SM, Rebbeck TR, White PS, Sulman EP, Jensen SJ (1997). Molecular genetic analysis of familial neuroblastoma. Eur J Cancer.

[18] Li S, Fell SM, Surova O, Smedler E, Wallis K, Chen ZX (2016). The 1p36 Tumor Suppressor KIF 1Bβ Is Required for Calcineurin Activation, Controlling Mitochondrial Fission and Apoptosis. Dev Cell.

[19] Choo Z, Koh RY, Wallis K, Koh TJ, Kuick CH, Sobrado V (2016). XAF1 promotes neuroblastoma tumor suppression and is required for KIF1Bβ-mediated apoptosis. Oncotarget.

[20] Chen ZX, Wallis K, Fell SM, Sobrado VR, Hemmer MC, Ramsköld D (2014). RNA helicase A is a downstream mediator of KIF1Bβ tumor-suppressor function in neuroblastoma. Cancer Discov.

[21] Fransson S, Martinsson T, Ejeskär K (2007). Neuroblastoma tumors with favorable and unfavorable outcomes: Significant differences in mRNA expression of genes mapped at 1p36.2. Genes Chromosomes Cancer..

[22] Yeh IT, Lenci RE, Qin Y, Buddavarapu K, Ligon AH, Leteurtre E (2008). A germline mutation of the KIF1B beta gene on 1p36 in a family with neural and nonneural tumors. Hum Genet.

[23] Guzmán C, Bagga M, Kaur A, Westermarck J, Abankwa D (2014). ColonyArea: an ImageJ plugin to automatically quantify colony formation in clonogenic assays. PLoS ONE.

[24] Berger AH, Knudson AG, Pandolfi PP (2011). A continuum model for tumour suppression. Nature..

[25] Henrich KO, Schwab M, Westermann F (2012). 1p36 tumor suppression–a matter of dosage?. Cancer Res.

[26] Bagchi A, Papazoglu C, Wu Y, Capurso D, Brodt M, Francis D (2007). CHD5 is a tumor suppressor at human 1p36. Cell.

[27] Schlisio S, Kenchappa RS, Vredeveld LC, George RE, Stewart R, Greulich H (2008). The kinesin KIF1Bbeta acts downstream from EglN3 to induce apoptosis and is a potential 1p36 tumor suppressor. Genes Dev.

[28] Nagai M, Ichimiya S, Ozaki T, Seki N, Mihara M, Furuta S (2000). Identification of the full-length KIAA0591 gene encoding a novel kinesin-related protein which is mapped to the neuroblastoma suppressor gene locus at 1p36.2. Int J Oncol..

[29] Bagchi A, Mills AA (2008). The quest for the 1p36 tumor suppressor. Cancer Res.

[30] Okawa ER, Gotoh T, Manne J, Igarashi J, Fujita T, Silverman KA (2008). Expression and sequence analysis of candidates for the 1p36.31 tumor suppressor gene deleted in neuroblastomas. Oncogene..

[31] Gorringe KL, Choong DY, Williams LH, Ramakrishna M, Sridhar A, Qiu W (2008). Mutation and methylation analysis of the chromodomain-helicase-DNA binding 5 gene in ovarian cancer. Neoplasia.

[32] Virden RA, Thiele CJ, Liu Z (2012). Characterization of critical domains within the tumor suppressor CASZ1 required for transcriptional regulation and growth suppression. Mol Cell Biol.

[33] Liu Z, Lam N, Wang E, Virden RA, Pawel B, Attiyeh EF (2017). Identification of CASZ1 NES reveals potential mechanisms for loss of CASZ1 tumor suppressor activity in neuroblastoma. Oncogene.

[34] Thompson PM, Gotoh T, Kok M, White PS, Brodeur GM (2003). CHD5, a new member of the chromodomain gene family, is preferentially expressed in the nervous system. Oncogene.

[35] Garcia I, Mayol G, Rodríguez E, Suñol M, Gershon TR, Ríos J (2010). Expression of the neuron-specific protein CHD5 is an independent marker of outcome in neuroblastoma. Mol Cancer.

[36] Paul S, Kuo A, Schalch T, Vogel H, Joshua-Tor L, McCombie WR (2013). Chd5 requires PHD-mediated histone 3 binding for tumor suppression. Cell Rep.

[37] Robbins CM, Tembe WA, Baker A, Sinari S, Moses TY, Beckstrom-Sternberg S (2011). Copy number and targeted mutational analysis reveals novel somatic events in metastatic prostate tumors. Genome Res.

[38] Ng D, Yang XR, Tucker MA, Goldstein AM (2008). Mutation screening of CHD5 in melanoma-prone families linked to 1p36 revealed no deleterious coding or splice site changes. BMC Res Notes.

[39] Lang J, Tobias ES, Mackie R (2011). Preliminary evidence for involvement of the tumour suppressor gene CHD5 in a family with cutaneous melanoma. Br J Dermatol.

[40] Schramm A, Köster J, Assenov Y, Althoff K, Peifer M, Mahlow E (2015). Mutational dynamics between primary and relapse neuroblastomas. Nat Genet.

[41] Kolla V, Zhuang T, Higashi M, Naraparaju K, Brodeur GM (2014). Role of CHD5 in human cancers: 10 years later. Cancer Res.

[42] Munirajan AK, Ando K, Mukai A, Takahashi M, Suenaga Y, Ohira M (2008). KIF1Bbeta functions as a haploinsufficient tumor suppressor gene mapped to chromosome 1p36.2 by inducing apoptotic cell death. J Biol Chem..

[43] Welander J, Andreasson A, Juhlin CC, Wiseman RW, Bäckdahl M, Höög A (2014). Rare germline mutations identified by targeted next-generation sequencing of susceptibility genes in pheochromocytoma and paraganglioma. J Clin Endocrinol Metab.

[44] Ma X, Li M, Tong A, Wang F, Cui Y, Zhang X (2020). Genetic and Clinical Profiles of Pheochromocytoma and Paraganglioma: A Single Center Study. Front Endocrinol (Lausanne).

[45] Seo SH, Kim JH, Kim MJ, Cho SI, Kim SJ, Kang H (2020). Whole Exome Sequencing Identifies Novel Genetic Alterations in Patients with Pheochromocytoma/Paraganglioma. Endocrinol Metab (Seoul).

[46] Evenepoel L, Helaers R, Vroonen L, Aydin S, Hamoir M, Maiter D, et al. KIF1B and NF1 are the most frequently mutated genes in paraganglioma and pheochromocytoma tumors. Endocr Relat Cancer. 2017;24(8):L57–61. 10.1530/ERC-17-0061 28515046

[47] Pillai S, Gopalan V, Lo CY, Liew V, Smith RA, Lam AK (2017). Silent genetic alterations identified by targeted next-generation sequencing in pheochromocytoma/paraganglioma: A clinicopathological correlations. Exp Mol Pathol.

[48] Capasso M, Diskin SJ, Totaro F, Longo L, De Mariano M, Russo R (2013). Replication of GWAS-identified neuroblastoma risk loci strengthens the role of BARD1 and affirms the cumulative effect of genetic variations on disease susceptibility. Carcinogenesis.

[49] Cimmino F, Avitabile M, Diskin SJ, Vaksman Z, Pignataro P, Formicola D (2018). Fine mapping of 2q35 high-risk neuroblastoma locus reveals independent functional risk variants and suggests full-length BARD1 as tumor-suppressor. Int J Cancer.

[50] Shi J, Yu Y, Jin Y, Lu J, Zhang J, Wang H (2019). Functional Polymorphisms in. J Cancer.

[51] Chan SH, Chew W, Ishak NDB, Lim WK, Li ST, Tan SH (2018). Clinical relevance of screening checklists for detecting cancer predisposition syndromes in Asian childhood tumours. NPJ Genom Med.

[52] Latorre V, Diskin SJ, Diamond MA, Zhang H, Hakonarson H, Maris JM (2012). Replication of neuroblastoma SNP association at the BARD1 locus in African-Americans. Cancer Epidemiol Biomarkers Prev.

[53] Zhang R, Zou Y, Zhu J, Zeng X, Yang T, Wang F (2016). The Association between GWAS-identified BARD1 Gene SNPs and Neuroblastoma Susceptibility in a Southern Chinese Population. Int J Med Sci.

