# Supplementary material for: Mutations of 1p genes do not consistently abrogate tumor suppressor functions in 1p-intact neuroblastoma
Source: BMC Cancer. 2022 Jun 30;22:717. doi: 10.1186/s12885-022-09800-0 (PMC9245282; doi:10.1186/s12885-022-09800-0)
Supplement: Supplementary file 1 — Additional file 1: Supplementary Table 1. Overview of all mutational variants identified in this study. [file 12885_2022_9800_MOESM1_ESM.pdf]

Supplementary Table 1. Overview of all mutational variants identified in this study

Candidate variant							Variant effect prediction						
**Gene**	**Exon**	**DNA change**	**AA change**	**Case no**	**AF**	**Mutation type**	**FATHMM (score)**	**SIFT**	**Poly-phen**	**MAF (1000G)**	**COSMIC**	**dbSNP**	**Clinvar**
CASZ1(1)	11	c.G2464A	p.G822S	1415T	0.55	Germline	-	T	B	0.0072	-		
	16	c.3327_3328insT	p.V1110fs	226T	0.43	ND	F	-	-	-	-	rs372839245	VCV000717999
	6	c.C829T	p.R277W	594T	0.48	Germline	P (0.82)	D	D	0.0012	COSM1732796		
	11	c.T2473C	p.S825P	626T	0.28	Germline						rs3748686	
	6	c.C829T	p.R277W	863T	0.51	Germline	P (0.82)	D	D	0.0012	COSM1732796	rs3748686	
	14	c.G2947A	p.A983T	422T	0.58	Germline	-	T	P	-	-	rs762996858	
	11	c.T2473C	p.S825P	66T	0.28	Germline (31%)	-	D	D	-	-		
	11	c.G4087A	p.A1363T	1330T	0.53	Germline	-	D	D	-	-		
PTPN14(2)	13	c.2147_2149del	p.E716del	844T	0.63	Germline	F	-	-	-	-		
	13	c.C1463T	p.P488L	558T	0.47	Germline	P (0.99)	D	D	-	COSM1689820	rs144475962	
	15	c.A2770G	p.I924V	1415T	0.5	Germline	-	T	B	0.034	-		
	18	c.C3344T	p.P1115L	2063T	0.55	ND	P (0.98)	T	D	0.0032	COSM1581553	rs200340171	VCV000717438
	4	c.G424A	p.A142T	936T	0.95	Somatic	-	D	D	-	-		
	13	c.G1349T	p.R450L	126T	0.52	Germline	P (0.99)	D	D	-	COSM5978226	rs373748699	
	13	c.G1093T	p.A365S	2216T	0.5	Germline	-	T	B	-	-	rs376896570	
	4	c.C418T	p.R140W	1761T	0.5	Germline	-	T	D	-	-	rs533046202	
CHD5(2)	21	c.G3217A	p.G1073S	797T	0.53	Somatic	-	T	D	.	-		
	4	c.C470T	p.T157M	171T	0.4	Germline	-	D	P	0.003	-	rs117780546	

	4	c.C470T	p.T157M	2220T	0.31	Germline	-	D	P	0.003	-	rs117780546	
	2	c.G127C	p.E43Q	1073T	0.28	Germline	-	D	B	0.0004	-		
	5	c.A704G	p.Q235R	1557T	0.72	ND	-	T	B	-	-	rs754661119	
	31	c.4614_4615AC	p.S1539P	936T	0.97	Germline	F	-	-	-	-		
	26	c.G3960A	p.W1320X	1348T	0.37	Somatic	S	-	-	-	-		
KIF1B(3)	22	c.C2134T	p.Q712X	797T	0.42	Somatic	-	.	.	.	-		
	22	c.A2192G	p.N731S	1371T	0.47	Germline	-	D	D	0.0078	-	rs117525287	VCV000240957
	33	c.C3649T	p.P1217S	305T	0.37	Germline	-	T	B	0.0002	-	rs121908163	VCV000004661
	29	c.A3260G	p.Y1087C	2251T	0.44	Germline	-	D	D	0.04	-	rs2297881	VCV000291567
	37	c.C4005G	p.I1335M	2707T	0.38	Germline	-	D	P	-	-		
	12	c.A1075G	p.N359D	2106T	0.28	Somatic	-	D	D	-	-		
	22	c.A2192G	p.N731S	452T	0.48	Germline	-	D	D	0.0078	-		
	32	c.C3519G	p.F1173L	422T	0.59	Germline	-	T	B	-	-	rs117525287	VCV000240957
NTRK1(4)	13	c.G1609A	p.D537N	797T	0.5	Somatic	P (0.80)	D	B	-	-	rs755016431	VCV000873802
	14	c.T1697C	p.M566T	37T	0.58	Germline	-	T	B	-	-	rs55892037	VCV000706659
	8	c.G1114T	p.A372S	37T	0.43	Germline	N (0.42)	T	B	0.0002	COSM676355	rs754611476	
	2	c.T38C	p.V13A	1236T	0.5	ND	-	T	B	0.0002	-	rs199647144	
	10	c.C1220T	p.P407L	2227T	0.5	Germline	P (0.95)	D	B	-	COSM6121710	rs750799657	VCV000639576
	10	c.C1220T	p.P407L	2256T	0.48	Germline	P (0.95)	D	B	-	COSM6121710	rs750799657	VCV000639576
BARD1(5, 6)	1	c.C34T	p.P12S	1371T	0.37	Somatic	-	T	P	-	-	rs1182316664	
	10	c.C1972T	p.R658C	249T	0.52	Germline	-	D	P	0.0054	-	rs3738888	VCV000136500
	4	c.1075_1095del	p.359_365del	2656T	0.41	Germline	F	-	-	-		rs28997575	
	1	c.G29A	p.R10Q	936T	0.25	Somatic	-	T	B	-	-		
	1	c.C34T	p.P12S	598T	0.4	ND	-	T	B	-	-	rs1182316664	
	7	c.G1670C	p.C557S	1348T	0.52	Germline	-	T	B	0.008	-		VCV000008045
	1	c.C35T	p.P12L	1660T	0.28	Somatic	-	T	B	-	-	rs786203647	VCV000187331

	1	c.T41A	p.I14N	817T	0.23	Germline (25%)	-	D	B	-	-	rs1574869913	
	1	c.T41A	p.I14N	141T	0.28	Germline (16%)	-	D	B	-	-	rs1574869913	
ALK(7)	16	c.G2672A	p.W891X	797T	0.3	Somatic	S	.	.	.			
	14	c.G2443A	p.A815T	797T	0.3	Somatic	-	T	D	.	-		
	5	c.G1202A	p.R401Q	797T	0.37	Somatic	P (0.91)	T	P	0.0002	COSM50278	rs188859061	VCV000663434
	29	c.A4573G	p.K1525E	525T	0.48	Germline	N (0.30)	T	B	0.001	COSM4592037		VCV000133475
	18	c.C3035T	p.T1012M	171T	0.51	Germline	-	T	P	0.002	-	rs35073634	VCV000133465
	25	c.G3824A	p.R1275Q	108T	0.25	Somatic	P (0.98)	D	D	-	COSM28056	rs113994087	VCV000018083
	25	c.G3824A	p.R1275Q	558T	0.37	Somatic	P (0.98)	D	D	-	COSM28056	rs113994087	VCV000018083
	21	c.3374_3375AA		518T	0.96	Somatic	F	-	-	-			
	25	c.G3824A	p.R1275Q	1807T	0.4	ND	P (0.98)	D	D	-	COSM28056	rs113994087	VCV000018083
	1	c.G357T	p.E119D	2063T	0.53	ND	-	T	B	-	-	rs758250431	VCV000538246
	23	c.T3521G	p.F1174C	2707T	0.19	Somatic	P (0.99)	D	D	-	COSM28059		VCV000375887
	13	c.C2210T	p.S737L	2707T	0.56	Germline	P (0.99)	T	D	-	COSM2941511	rs368581969	VCV000335703
	1	c.G386T	p.G129V	2707T	0.32	Germline (44%)	-	D	B	-	-	rs760041708	VCV000335714
	23	c.C3522A	p.F1174L	2106T	0.18	Somatic	P (0.99)	D	P	-	COSM28055	rs863225281	VCV000217851
	14	c.G2431A	p.V811M	1707T	0.5	ND	P (0.81)	T	D	-	COSM145089	rs771935814	VCV000836307
	29	c.C4587A	p.D1529E	936T	0.9	Germline	N (0.00)	T	B	-	COSM3758201		
	1	c.G487T	p.V163L	936T	0.42	Germline	-	T	B	0.0012	-	rs55697431	VCV000133459
	22	c.G3481A	p.E1161K	1660T	0.5	Germline	-	D	D	-	-	rs145194836	VCV000569028
	1	c.C409G	p.R137G	2771T	0.86	ND	-	D	D	-	-	rs757382067	VCV000538185
	25	c.G3824A	p.R1275Q	1584T	0.52	Germline (50%)	P (0.98)	D	D	-	COSM28056	rs113994087	VCV000018083
	7	c.C1486A	p.P496T	1584T	0.52	Germline	-	T	B	-	-		
	23	c.C3522A	p.F1174L	748T	0.48	Somatic	P (0.99)	D	P	-	COSM28055	rs863225281	VCV000217851
	23	c.C3522A	p.F1174L	590T	0.43	Somatic	P (0.99)	D	P	-	COSM28055	rs863225281	VCV000217851
PHOX2B(8)	3	c.G456C	p.K152N	2063T	0.48	ND	-	D	P	-	-		

	2	c.C315A	p.F105L	1660T	0.56	Germline	-	D	D	-	-		
LIN28B(7)	4	c.G586A	p.G196R	2220T	0.64	Germline	P (0.94)	D	D	-	COSM3619146		
	4	c.C614T	p.P205L	1660T	0.47	Germline	-	T	B	0.0002	-		
NTRK2(4)	14	c.G1392T	p.M464I	95T	0.39	Somatic	P (0.87)	T	P	-	COSM5064125		
	4	c.A211G	p.I71V	2216T	0.48	Germline	-	T	D	-	-		
PTPN11(7)	13	c.G1507C	p.G503R	166T	0.2	Somatic	P (0.99)	D	D	-	COSM4384772	rs397507545	VCV000040558
BRCA2(6)	18	c.G8092A	p.A2698T	1371T	0.52	Germline	N (0.00)	T	B	-	COSM3399345	rs80359052	VCV000038138
	11	c.C2918T	p.S973L	770T	0.5	Germline	-	D	B	-	-	rs397507296	VCV000037807
	18	c.G8092A	p.A2698T	305T	0.35	Germline	N (0.00)	T	B	-	COSM3399345		VCV000038138
	18	c.G8187T	p.K2729N	2220T	0.34	Germline (44%)	P (0.88)	D	D	0.0026	COSM5021110	rs80359065	VCV000038142
	11	c.G2573C	p.R858T	2707T	0.48	Germline	N (0.00)	T	B	-	COSM7113616		
	10	c.T943A	p.C315S	1557T	0.47	ND	n/a	T	B	0.0016	COSM7002405	rs79483201	VCV000038241
	11	c.G4109C	p.G1370A	598T	0.47	ND	-	T	B	-	-	rs587781406	VCV000186065
	14	c.T7102G	p.L2368V	452T	0.49	Germline	-	T	B	-	-		VCV000038084
	11	c.C4178T	p.A1393V	2130T	0.46	Germline	N (0.07)				COSM4047070	rs398122776	VCV000089047
	4	c.A386T	p.D129V	404T	0.68	Germline (53%)	-	D	D	-	-		
	11	c.C3453G	p.I1151M	404T	0.68	Germline (50%)	-	D	P	-	-		VCV000037842
TIAM1(9)	29	c.G4616A	p.G1539D	844T	0.5	Germline	-	D	B	0.0046	-	rs34393355	
	17	c.A2893T	p.S965C	2011T	0.51	ND	-	T	P	0.0014	-	rs373619112	
	17	c.A2893T	p.S965C	1120T	0.48	ND	-	T	P	0.0014	-	rs373619112	
	19	c.G3235A	p.D1079N	2656T	0.51	Germline	-	D	P	-	-		
	17	c.C2966T	p.S989L	1491T	0.49	Germline	-	T	B	-	-	rs753290841	
ATRX(9)	35	c.C7462T	p.Q2488X	797T	0.27	Somatic		.	.	.	(stop codon)		
	9	c.T2540C	p.F847S	797T	0.61	Germline	N (0.10)	T	B	0.0045	COSM7238259	rs45624939	VCV000133660
	9	c.G1948A	p.E650K	797T	0.26	Somatic	-	D	P	.	-		
	9	c.A2701G	p.I901V	280T	0.47	Germline	-	T	B	0.0005	-	rs587778087	VCV000133651

	9	c.T3345A	p.Y1115X	1761T	0.62	Somatic		-	-	-	(stop codon)		
	9	c.1989delC	p.L664fs*0	748T	0.93	Somatic		-	-	-	(frameshift)		
	9	c.G2806C	p.V936L	668T	1	Germline	-	T	B	0.0008	-	rs149232501	VCV000128496
	9	c.A3646G	p.I1216V	1807T	0.47	ND	N (0.05)	T	B	0.0003	COSM3097071	rs782062542	VCV000796709
	9	c.T2540C	p.F847S	2072T	1	Germline	N (0.10)	T	B	0.0045	COSM7238259	rs45624939	VCV000133660
	9	c.T2540C	p.F847S	1726T	0.5	Germline	N (0.10)	T	B	0.0045	COSM7238259	rs45624939	VCV000133660
	9	c.T2540C	p.F847S	863T	1	Germline	N (0.10)	T	B	0.0045	COSM7238259	rs45624939	VCV000133660

AF: allelic frequency, P: pathogenic, T: tolerated, D: deleterious, B: benign, F: frameshift, N: neutral, S: stop codon, ND: not determined

References

1. Carén H, Fransson S, Ejeskär K, Kogner P, Martinsson T. Genetic and epigenetic changes in the common 1p36 deletion in neuroblastoma tumours. Br J Cancer. 2007;97(10):1416-24.
2. Schramm A, Köster J, Assenov Y, Althoff K, Peifer M, Mahlow E, et al. Mutational dynamics between primary and relapse neuroblastomas. Nat Genet. 2015;47(8):872-7.
3. Carén H, Ejeskär K, Fransson S, Hesson L, Latif F, Sjöberg RM, et al. A cluster of genes located in 1p36 are down-regulated in neuroblastomas with poor prognosis, but not due to CpG island methylation. Mol Cancer. 2005;4(1):10.
4. Light JE, Koyama H, Minturn JE, Ho R, Simpson AM, Iyer R, et al. Clinical significance of NTRK family gene expression in neuroblastomas. Pediatr Blood Cancer. 2012;59(2):226-32.
5. Capasso M, Devoto M, Hou C, Asgharzadeh S, Glessner JT, Attiyeh EF, et al. Common variations in BARD1 influence susceptibility to high-risk neuroblastoma. Nat Genet. 2009;41(6):718-23.
6. Fransson S, Martinez-Monleon A, Johansson M, Sjöberg RM, Björklund C, Ljungman G, et al. Whole-genome sequencing of recurrent neuroblastoma reveals somatic mutations that affect key players in cancer progression and telomere maintenance. Sci Rep. 2020;10(1):22432.
7. Pugh TJ, Morozova O, Attiyeh EF, Asgharzadeh S, Wei JS, Auclair D, et al. The genetic landscape of high-risk neuroblastoma. Nat Genet. 2013;45(3):279-84.

8. Raabe EH, Laudenslager M, Winter C, Wasserman N, Cole K, LaQuaglia M, et al. Prevalence and functional consequence of PHOX2B mutations in neuroblastoma. Oncogene. 2008;27(4):469-76.
9. Molenaar JJ, Koster J, Zwijnenburg DA, van Sluis P, Valentijn LJ, van der Ploeg I, et al. Sequencing of neuroblastoma identifies chromothripsis and defects in neuritogenesis genes. Nature. 2012;483(7391):589-93.
