# Supplementary material for: Mutations of 1p genes do not consistently abrogate tumor suppressor functions in 1p-intact neuroblastoma
Source: BMC Cancer. 2022 Jun 30;22:717. doi: 10.1186/s12885-022-09800-0 (PMC9245282; doi:10.1186/s12885-022-09800-0)
Supplement: Supplementary file 2 — Additional file 2: Supplementary Table 2. Novel variants not previously described in dbSNP, Clinvar, 1000G, COSMIC databases. [file 12885_2022_9800_MOESM2_ESM.docx]

**Supplementary Table 2. Novel variants not previously described in dbSNP, Clinvar, 1000G, COSMIC databases**

| **Candidate variant** | | | | **Variant effect** | **Cases** | **AF** | **Mutation type** | **Variant effect predictions** | | |
| --- | --- | --- | --- | --- | --- | --- | --- | --- | --- | --- |
| **Gene** | **Exon** | **DNA change** | **AA change** |  |  |  |  | **FATHMM** | **SIFT** | **Polyphen** |
| CASZ1 | 20 | c.G4087A | p.A1363T | nonsynonymous SNV | 1330T | 0.53 | Germline | - | D | D |
|  | 16 | c.3327_3328insT | p.V1110fs | frameshift insertion | 226T | 0.43 | ND | (Frameshift -non-T polymer) | - | - |
|  | 11 | c.T2473C | p.S825P | nonsynonymous SNV | 66T | 0.28 | Germline (31%) | - | D | D |
|  | 11 | c.T2473C | p.S825P | nonsynonymous SNV | 626T | 0.28 | Germline (38%) | - | D | D |
| PTPN14 | 13 | c.2147_2149del | p.E716del | nonframeshift deletion | 844T | 0.63 | Germline | (Frameshift ) | - | - |
|  | 4 | c.G424A | p.A142T | nonsynonymous SNV | 936T | 0.95 | Somatic | - | D | D |
| CHD5 | 2 | c.G127C | p.E43Q | nonsynonymous SNV | 1073T | 0.28 | Germline | - | D | B |
|  | 21 | c.G3217A | p.G1073S | nonsynonymous SNV | 797T | 0.53 | Somatic | - | T | D |
|  | 31 | c.4614_4615AC | p.S1539P | nonframeshift substitution | 936T | 0.97 | Germline | (Frameshift ) | - | - |
|  | 26 | c.G3960A | p.W1320X | stopgain | 1348T | 0.37 | Somatic | Stop codon | - | - |
| KIF1B | 22 | c.C2134T | p.Q712X | stopgain | 797T | 0.42 | Somatic | - | . | . |
|  | 37 | c.C4005G | p.I1335M | nonsynonymous SNV | 2707T | 0.38 | Germline | - | D | P |
|  | 12 | c.A1075G | p.N359D | nonsynonymous SNV | 2106T | 0.28 | Somatic | - | D | D |
| BARD1 | 1 | c.G29A | p.R10Q | nonsynonymous SNV | 936T | 0.25 | Somatic | - | T | B |
| ALK | 14 | c.G2443A | p.A815T | nonsynonymous SNV | 797T | 0.3 | Somatic | - | T | D |
|  | 7 | c.C1486A | p.P496T | nonsynonymous SNV | 1584T | 0.52 | Germline | - | T | B |
| PHOX2B | 3 | c.G456C | p.K152N | nonsynonymous SNV | 2063T | 0.48 | ND | - | D | P |
|  | 2 | c.C315A | p.F105L | nonsynonymous SNV | 1660T | 0.56 | Germline | - | D | D |
| NTRK2 | 4 | c.A211G | p.I71V | nonsynonymous SNV | 2216T | 0.48 | Germline | - | T | D |
| BRCA2 | 4 | c.A386T | p.D129V | nonsynonymous SNV | 404T | 0.68 | Germline(53%) | - | D | D |
| TIAM1 | 19 | c.G3235A | p.D1079N | nonsynonymous SNV | 2656T | 0.51 | Germline | - | D | P |
| ATRX | 35 | c.C7462T | p.Q2488X | stopgain | 797T | 0.27 | Somatic |  | . | . |
|  | 9 | c.G1948A | p.E650K | nonsynonymous SNV | 797T | 0.26 | Somatic | - | D | P |
|  | 9 | c.T3345A | p.Y1115X | stopgain | 1761T | 0.62 | Somatic |  | - | - |
|  | 9 | c.1989delC | p.L664fs*0 | frameshift deletion | 748T | 0.93 | Somatic |  | - | - |

AF: allelic frequency, P: pathogenic, T: tolerated, D: deleterious, B: benign, F: frameshift, N: neutral, S: stop codon, ND: not determined
