# Supplementary material for: Mutations of 1p genes do not consistently abrogate tumor suppressor functions in 1p-intact neuroblastoma
Source: BMC Cancer. 2022 Jun 30;22:717. doi: 10.1186/s12885-022-09800-0 (PMC9245282; doi:10.1186/s12885-022-09800-0)

Additional File 3

(A) SK-N-AS FLAG-tag full length blot (in Fig 5C)

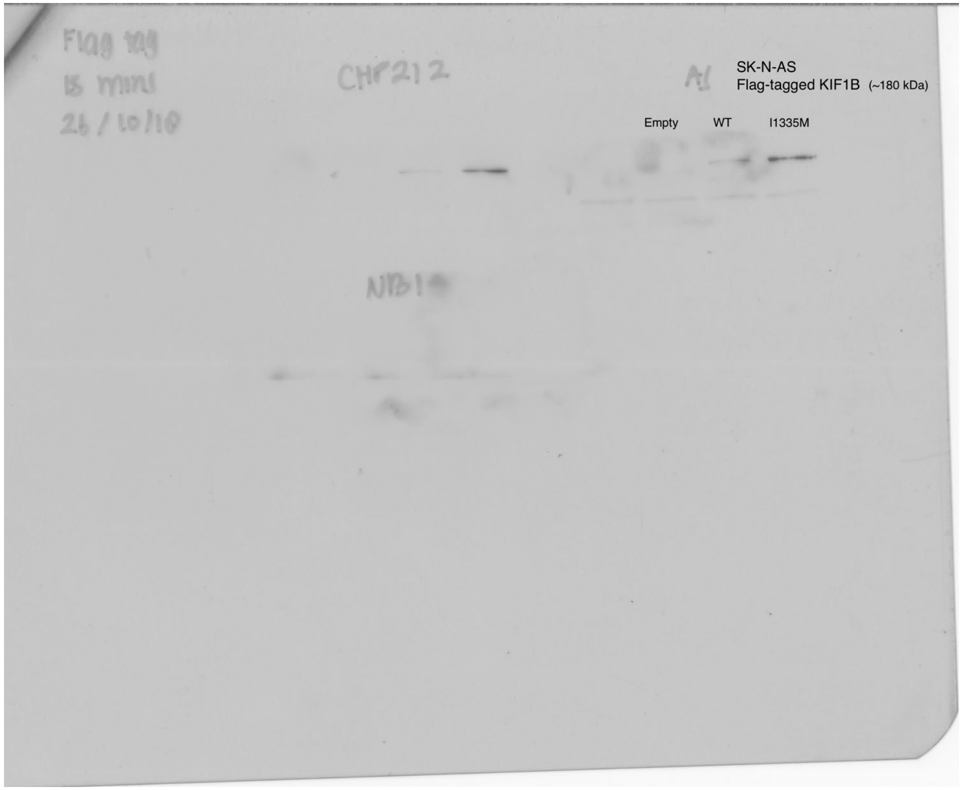

(B) SK-N-AS B-actin full length blot (in Fig 5C)

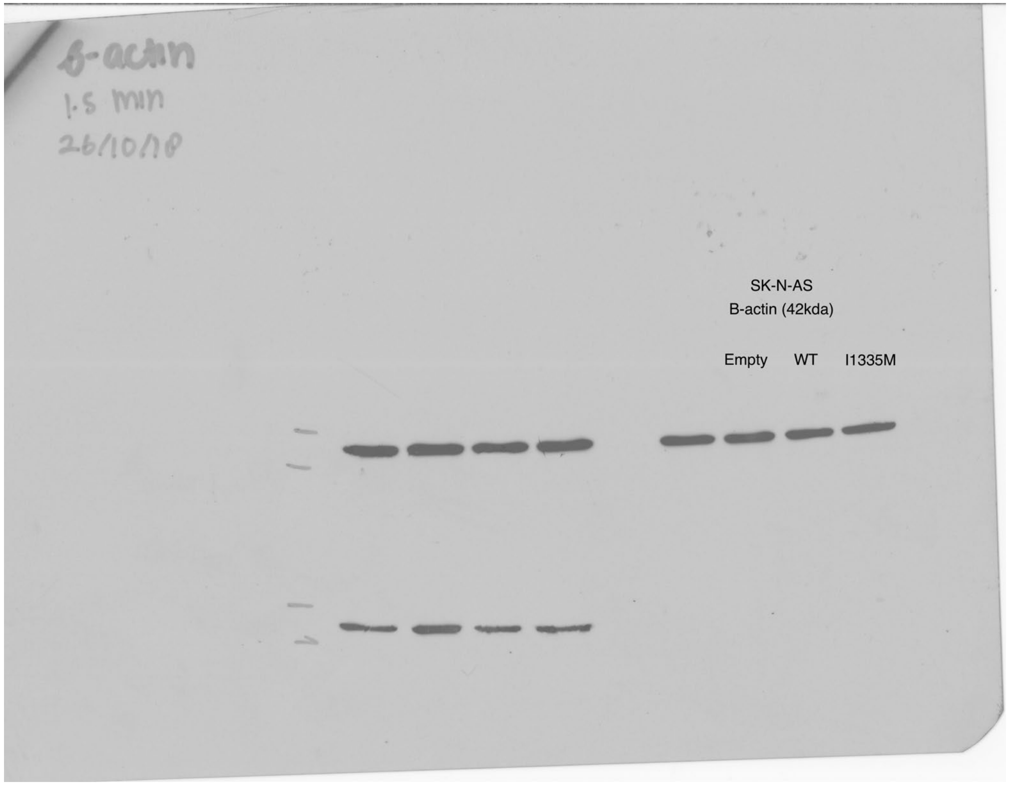

(C) CHP212 FLAG-tag full length blot (in Fig 5C)

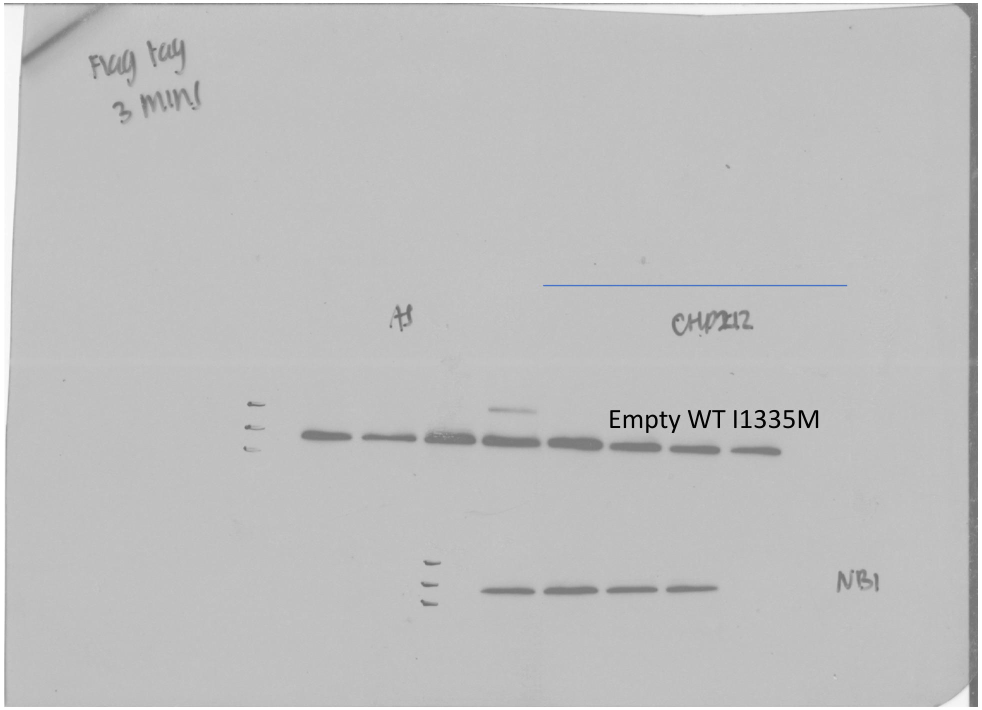

(D) CHP212 B-actin full length blot (in Fig 5C)

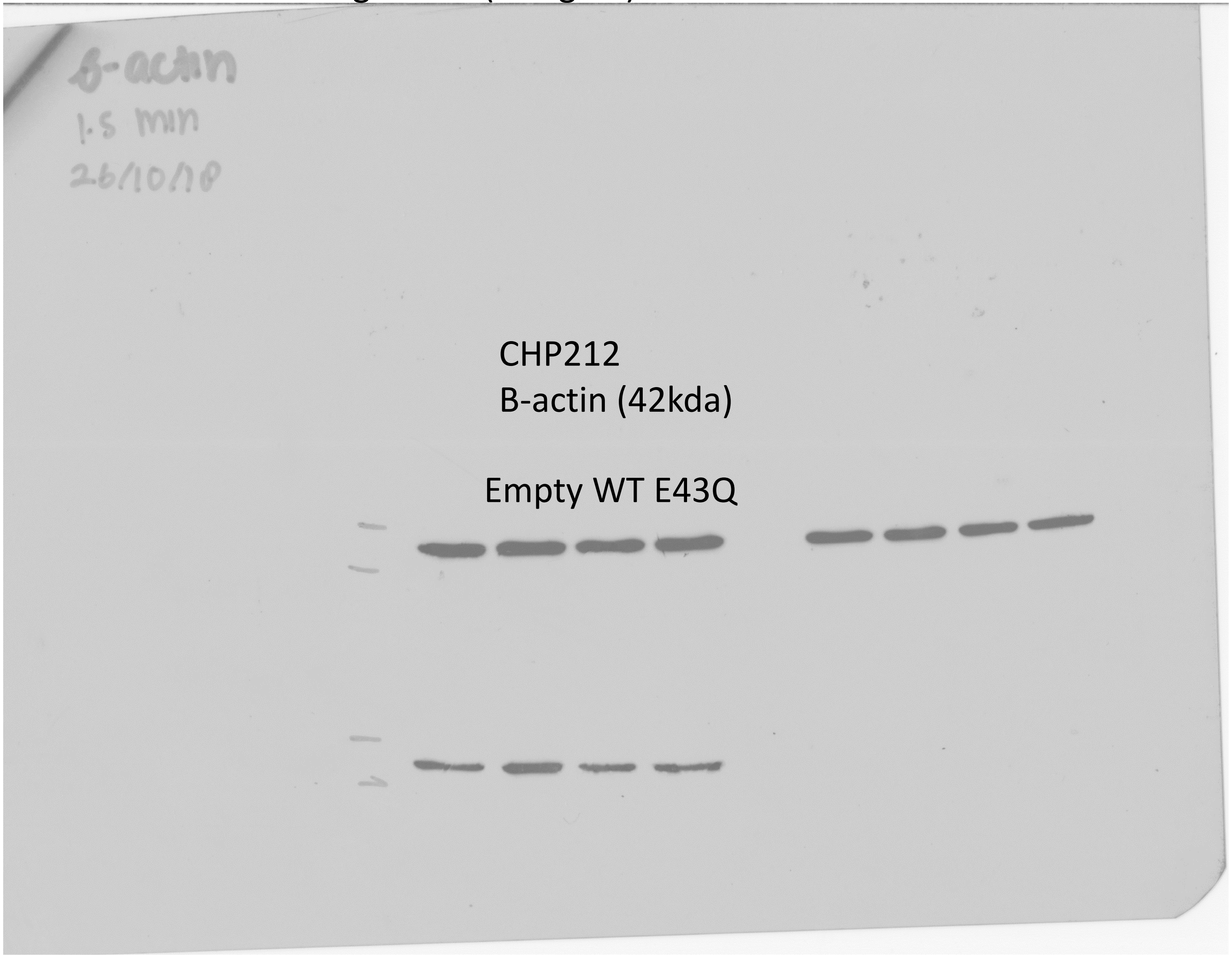

(E) SK-N-AS His-tag full length blot (in Fig 5G)

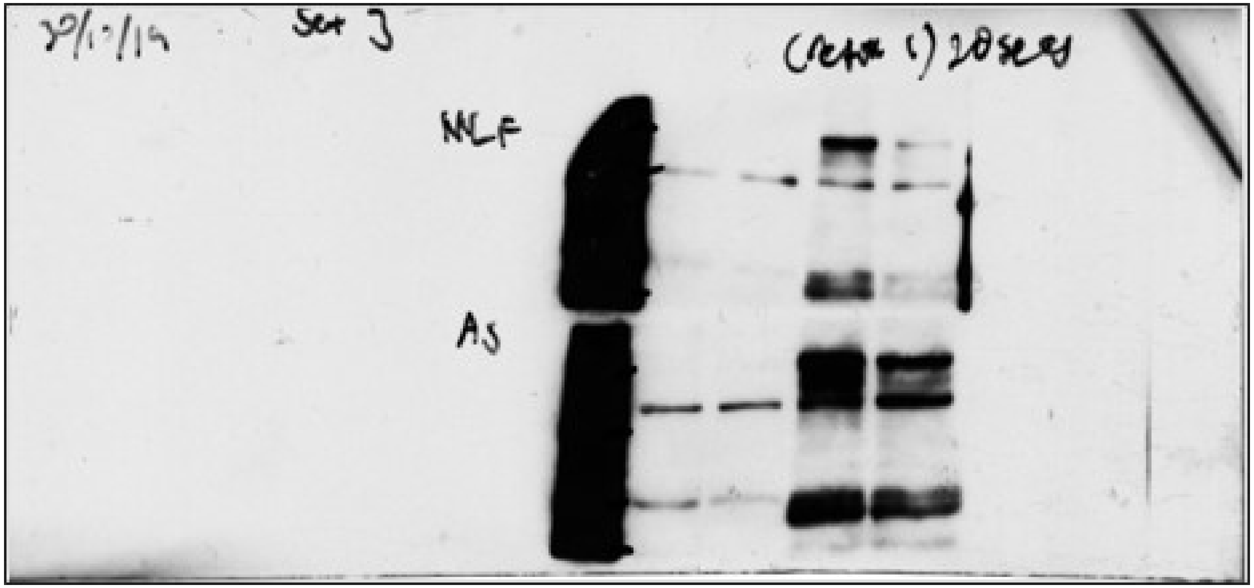

Mock Empty WT E43Q

(F) SK-N-AS B-actin full length blot (in Fig 5G)

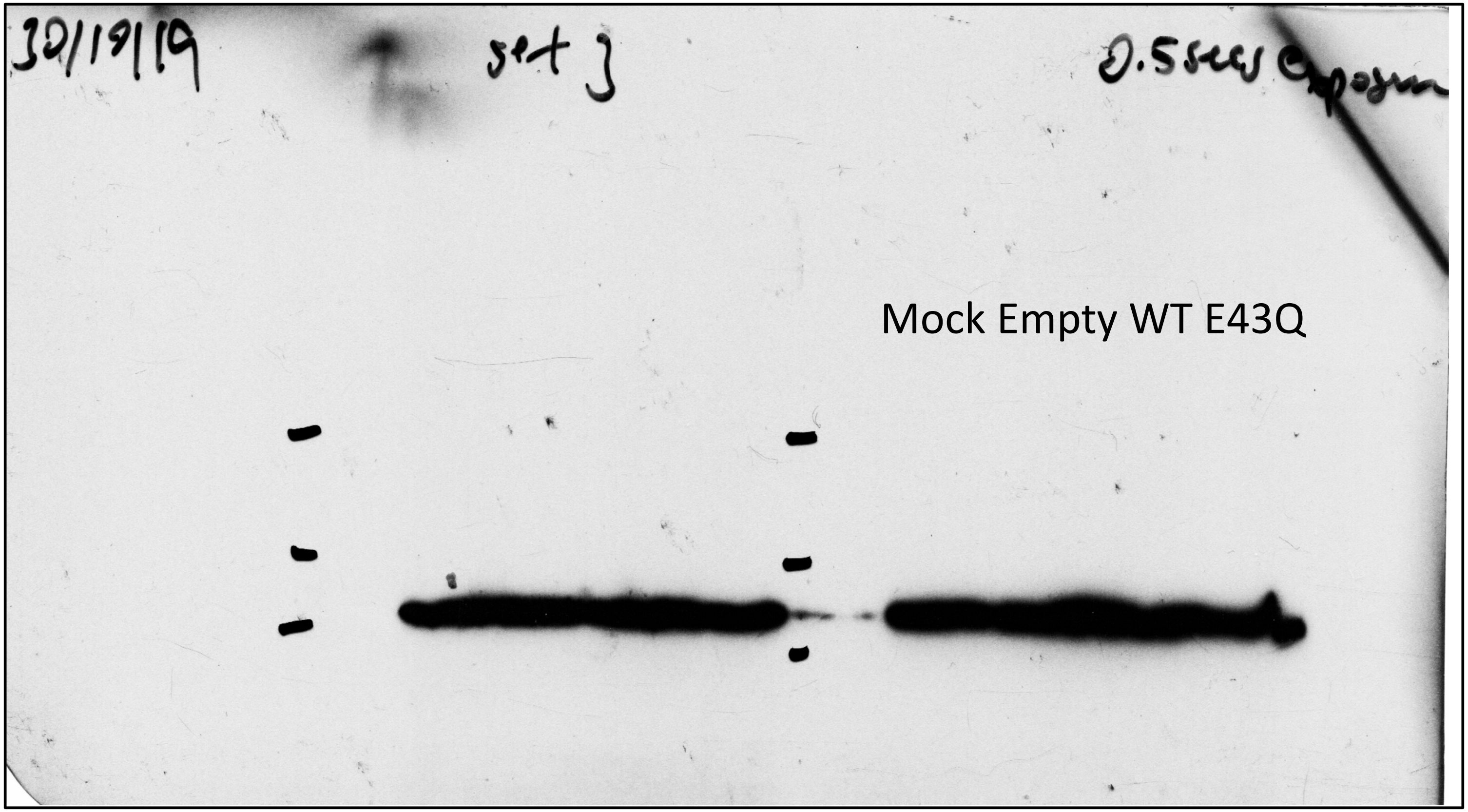

(G) CHP212 His-tag full length blot (in Fig 5G)

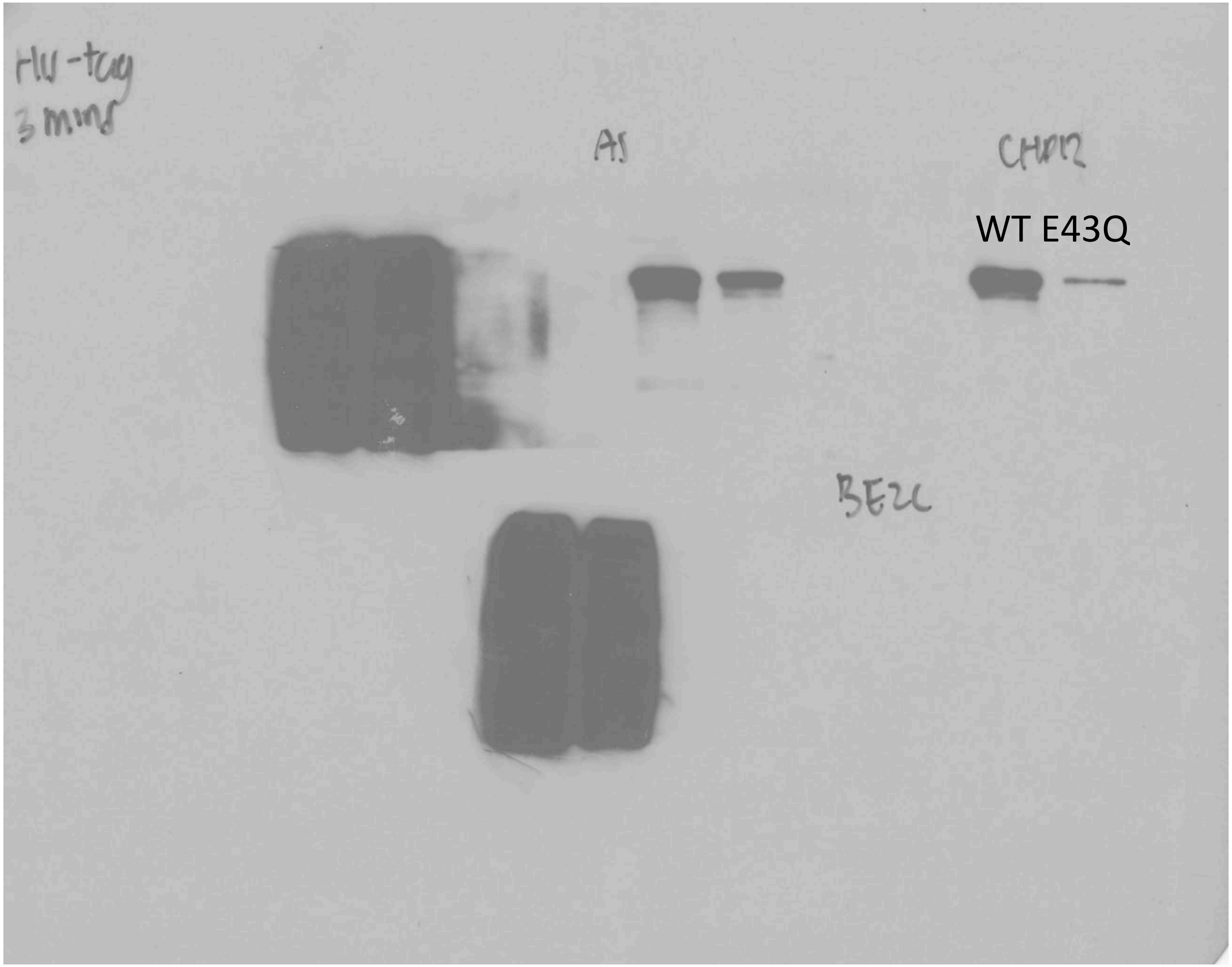

(H) CHP212 B-actin full length blot (in Fig 5G)

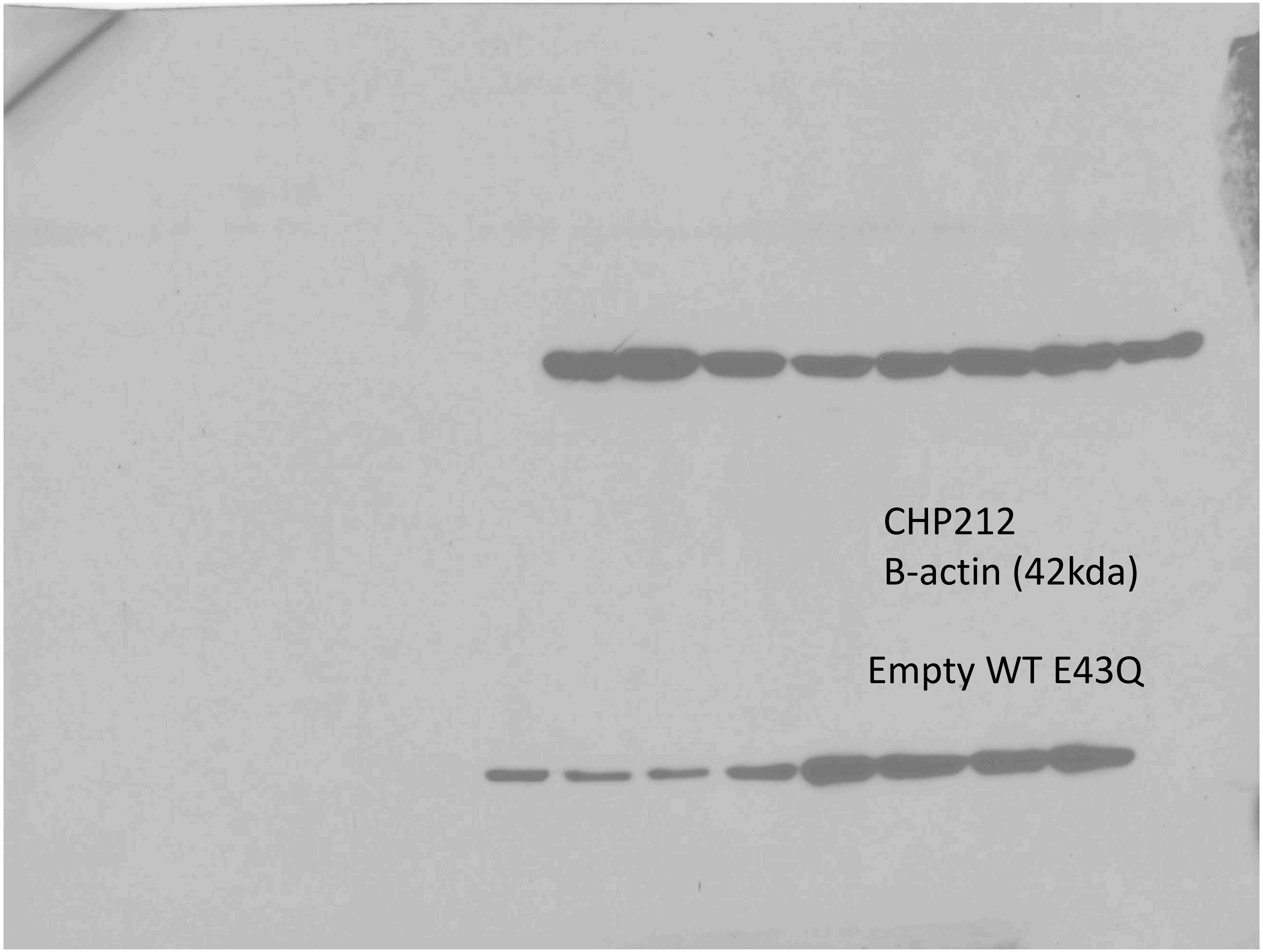

(I) NLF His-tag full length blot (in Fig 5G)

Mock Empty E43Q WT

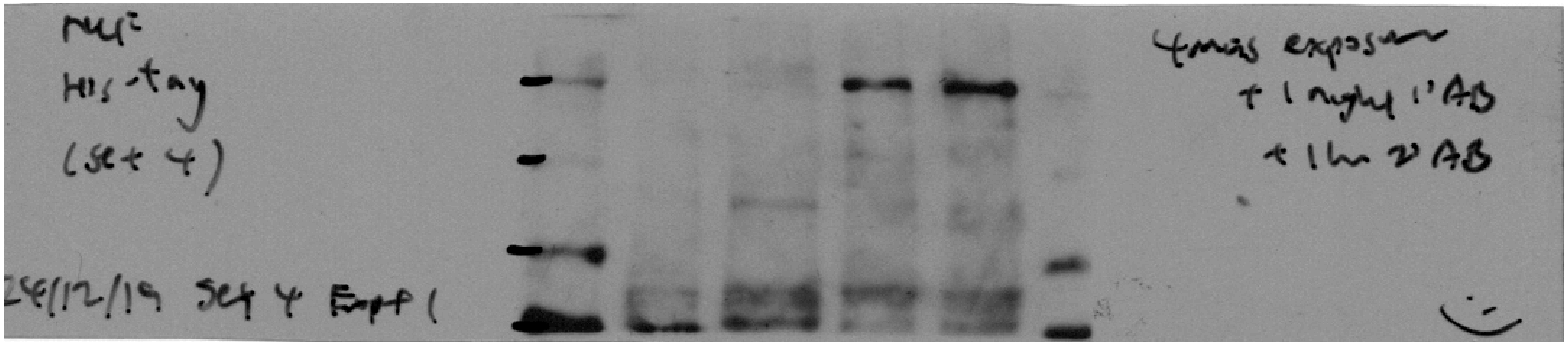

(J) NLF B-actin full length blot (in Fig 5G)

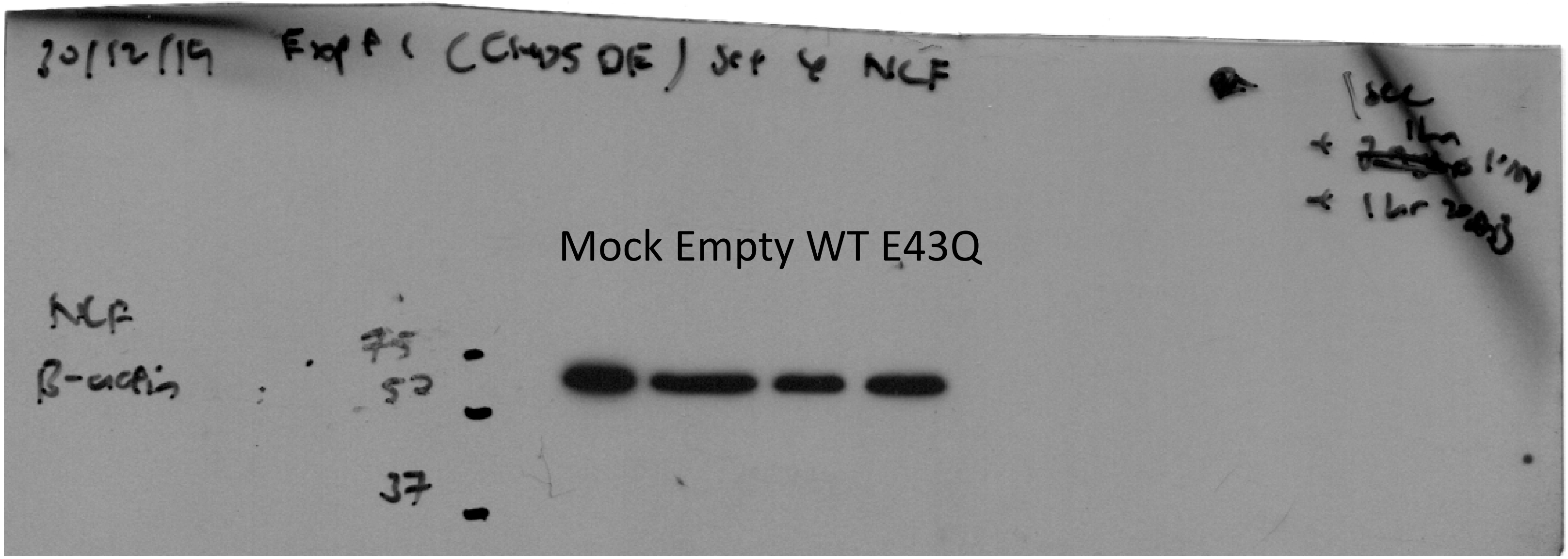

Supplement: Supplementary file 3 — Additional file 3. Full length western blots. [file 12885_2022_9800_MOESM3_ESM.pdf]
