# Supplementary material for: Mutations of 1p genes do not consistently abrogate tumor suppressor functions in 1p-intact neuroblastoma
Source: BMC Cancer. 2022 Jun 30;22:717. doi: 10.1186/s12885-022-09800-0 (PMC9245282; doi:10.1186/s12885-022-09800-0)
Supplement: Supplementary file 4 — Additional file 4. Supplementary methods. [file 12885_2022_9800_MOESM4_ESM.docx]

**Supplementary methods**

**Validation of variants with Sanger sequencing**

Confirmation of *CHD5* (NM_015557) c.127G>C p.E43Q and *KIF1B* (NM_015074) c.4005G>C: p.I1335M mutations were performed using primers: CHD5 F-CCTCACCTCTTTCTTCTTCCG; CHD5 R-TGGAGGAAGCTCAGGAGTAA and KIF1B F-TTTGAGGCTGTGTGGGATAG; KIF1B R-TGACCACTTCCTGCTGTAAC respectively. Sanger Sequencing on the amplicons were performed on a SeqStudio Sequencer (Applied Biosystems) to confirm the nucleotide changes.

**Cell line transfection**

In Opti-MEM Reduced Serum Medium, transfections of cell lines SK-N-AS and NLF neuroblastoma cell line were performed using 4μg of either empty pcDNA3.1, WT His-tagged CHD5 or E43Q mutant His-tagged CHD5 plasmids using Lipofectamine 2000 (Invitrogen) in a DNA:Lipo ratio of 1:1.25/well following manufacturer’s protocol. CHD5 plasmids were gifted by Dr. Kolla Venkatadri (Division of Oncology, Department of Paediatrics, The Children’s Hospital of Philadelphia, Philadelphia, U.S.A). E43Q mutant his-tagged CHD5 plasmids were generated using site-directed mutagenesis and colony PCR previously. Preparation of plasmid stocks was done following Maxiprep Kit and protocols (QIAGEN). After 24 hr post-transfection, cells were maintained in fresh medium. Cells were harvested 48 hr post-transfection for subsequent experiments.

**Lentiviral-delivered shRNA**

Using two shRNAs (Sigma Aldrich), two lentiviral short hairpin RNAs (shRNA) vectors, shCHD5#76 and shCHD5#95, were constructed via transfection in 293T cells using Lipofectamine 2000 (Invitrogen) and harvested at 48h and 72h post-transfection. 293FT cells were maintained in DMEM/High Glucose (Hyclone) with 10% FBS and 1% Sodium Pyruvate. Upon harvesting at 96 hr, virus-containing supernatants were removed and ultracentrifuged. Virus-containing pellet was resuspended in DMEM and stored in -80°C. The sequences used were as follows:

shCHD5#76:

5’– CCGGTGATAACCAGTCAGAATATTCCTCGAGGAATATTCTGACTGGTTATCATT TTTG–3’

shCHD5#95:

5’–

CCGGCCTGGAGA TGAAGAACAAGTTCTCGAGAACTTGTTCTTCA TCTCCAGGTT TTT–3’

**Cell line transduction**

Cell line SK-N-AS was transduced with the following shRNA-expressing lentiviral plasmids containing puromycin resistance gene for selection: scrambled shRNA (shSCR) (Sigma-Aldrich) as control (2μl), shCHD5#76 (2μl) and shCHD5#95 (1μl). At 24 hr post-transduction, the medium was changed with fresh medium supplemented with 1μg/ml puromycin (Gibco). These concentrations were determined from a previous optimisation that was carried out in the laboratory. Cells were harvested 96 hr post-transduction for subsequent experiments.
